# A Micropolymorphism Altering the Residue Triad 97/114/156 Determines the Relative Levels of Tapasin Independence and Distinct Peptide Profiles for HLA-A^*^24 Allotypes

**DOI:** 10.1155/2014/298145

**Published:** 2014-12-04

**Authors:** Soumya Badrinath, Heike Kunze-Schumacher, Rainer Blasczyk, Trevor Huyton, Christina Bade-Doeding

**Affiliations:** Institute for Transfusion Medicine, Hannover Medical School, Medical Park, Feodor Lynen Street 21, 30625 Hannover, Germany

## Abstract

While many HLA class I molecules interact directly with the peptide loading complex (PLC) for conventional loading of peptides certain class I molecules are able to present peptides in a way that circumvents the PLC components. We investigated micropolymorphisms at position 156 of HLA-A^*^24 allotypes and their effects on PLC dependence for assembly and peptide binding specificities. HLA-A^*^24:06^156Trp^ and HLA-A^*^24:13^156Leu^ showed high levels of cell surface expression while HLA-A^*^24:02^156Gln^ was expressed at low levels in tapasin deficient cells. Peptides presented by these allelic variants showed distinct differences in features and repertoire. Immunoprecipitation experiments demonstrated all the HLA-A^*^24/156 variants to associate at similar levels with tapasin when present. Structurally, HLA-A^*^24:02 contains the residue triad Met97/His114/Gln156 and a Trp156 or Leu156 polymorphism provides tapasin independence by stabilizing these triad residues, thus generating an energetically stable and a more peptide receptive environment. Micropolymorphisms at position 156 can influence the generic peptide loading pathway for HLA-A^*^24 by altering their tapasin dependence for peptide selection. The trade-off for this tapasin independence could be the presentation of unusual ligands by these alleles, imposing significant risk following hematopoietic stem cell transplantation (HSCT).

## 1. Introduction

Allogeneic hematopoietic stem cell transplantation (HSCT) is an established treatment of various haematological malignancies and certain congenital metabolic disorders. The chance of finding a human leucocyte antigen- (HLA-) identical donor-recipient pair amongst siblings is only one in four. Therefore, stem cells from unrelated donors are most frequently used for transplantation [1]. However, these transplants are associated with significant risks of Graft versus Host Disease (GvHD), graft failure, and transplant related mortality [2]. The magnitude of permissivity of a given class I allotype is found to depend on the position of mismatched amino acids (AA) within the class I heavy chain (hc) and nature of the exchanged AA and also of the neighbouring AAs [3]. The mismatched AA might impact the peptides that are selected and presented by these individual class I allotypes and also alter the conformation of these peptide-HLA (pHLA) complexes [4–7]. Previous studies have shown that even a single AA exchange occurring at key residues within the peptide binding groove can strongly influence the features of bound peptides [8] and result in severe incompatibility [9]. It is therefore of crucial importance to determine the most acceptable mismatch when no identical matched donor is available. This can be achieved by conducting a systematic study to understand the effect of AA polymorphisms occurring in HLA class I molecules.

HLA class I molecules play an important role in binding and presentation of high affinity peptides to the cell surface for its subsequent recognition by CD8^+^ T-cells [10]. For its efficient cell surface expression, the class I hc must associate with beta-2-microglobulin (b2m) and cytosolically derived peptides within the endoplasmic reticulum (ER), a process involving several components of the peptide loading complex (PLC) [11]. Calnexin assists in proper folding of class I hc prior to its association with b2m and thereafter with the chaperon calreticulin (CRT) [10]. The transporter associated with antigen processing (TAP) facilitates the import of proteasomally processed peptides from cytosol into the ER [12]. The transmembrane glycoprotein tapasin (TPN) functions within the PLC as a stable disulphide linked heterodimer along with ERp57 [13]. TPN bridges the CRT-class I/b2m complex to the transporter associated with antigen processing (TAP) [14], thereby facilitating peptide loading. Additionally, TPN is also found to increase the steady state levels of TAP [10] and help in the optimization and loading of high affinity peptides.

HLA class I polymorphisms primarily concentrated in the peptide binding region (PBR) not only influence the features and repertoire of peptides presented to the cell surface but also affect their requirement on the PLC components for antigen processing and presentation [15–19]. Most of the HLA class I allotypes such as HLA-B8 [17], HLA-B^*^44:02 [17, 20], and HLA- B^*^15:10 [18] are highly dependent upon TPN for peptide loading and antigen presentation [17, 21]. Other alleles such as HLA-B^*^27:05 [17], HLA-B^*^44:05 [16], HLA-B^*^15:01, and HLA-B^*^15:18 [18] were shown to load peptides without TPN, though most of these allotypes associate with the PLC and make use of TPN when present [16, 17, 22]. We recently described position 156 located at the centre of the *α*2 helix within the HLA class I hc to be an important determinant for its association with TPN. The great impact of position 156 on HLA function is reflected by its large HLA class I polymorphism and its influence in peptide binding. By structural analysis residue 156 could be described to contact position 3 of a bound peptide in almost all structures (>70%) and position 4, 5, 6, or 7 in 5–70% of peptide-HLA structures available [8]. Even a single mismatch at position 156 can result in transplant rejection [9, 23] and acute GvHD for HLA-B^*^44 allotypes [24]. Structural involvement of position 156 in influencing the conformation of PBR was demonstrated by comparing the crystal structures of HLA-B^*^44:02^156Asp^ and B^*^44:03^156Leu^ complexed with the same, natural high affinity ligand [25]. Recently, we described the effect of single AA polymorphisms at position 156 on TPN dependency for HLA-B^*^44 allotypes for optimal peptide loading and cell surface expression. Our results illustrated that TPN independent peptide loading resulted in the presentation of exclusive set of peptides with distinct features and lengths [6]. TPN independent alleles are able to load and present peptides via a nonclassical peptide loading pathway and for that reason TPN independent alleles should be able to overcome virus escape mechanism of blocking TPN and might be able to present viral peptides. TPN independence offers flexibility on one hand, because it provides an effective pathogen evasion; however peptides are loaded suboptimally and that might influence the immunogenicity and half-life time of pHLA complexes and this might result in autoimmunity.

One of the most common occurring HLA class I variants worldwide is HLA-A^*^24:02 (http://www.allelefrequencies.net); within the HLA-A^*^24 group are 3 naturally occurring variants that differ exclusively at residue 156: HLA-A^*^24:02^156Gln^, A^*^24:06^156Trp^, and A^*^24:13^156Leu^. Since HLA-A^*^24:02 was described to process and present the immediate early-1 antigen (IE-1) derived peptides of HCMV [26], it becomes obvious that HLA-A^*^24:02 is able to load peptides independently of the PLC; however the presentation of viral peptides seems to be orchestrated by the interaction of HLA and HCMV immune evasions [27]. Peptides from IE-1 are found to be presented 6 hours after infection, though it has been shown that the presentation of different IE1 epitopes is suppressed strongly after infection with US2/US11-competent strains, but not after infection with US2/US11-deleted viral strains (between 1 and 91 hours after infection). The deletion of US2, US6, and US11 enabled the HLA molecules to restore the presentation of IE-1 peptides to CTLs [28]. However, US3 deletion had no effect on IE-1 suppression, suggesting that the increased retention times of HLA class I molecules in the ER by US3 in TPN dependent allotypes results in the exchange of IE-1 peptides with higher affinity self-peptides. However, given that HLA-A^*^24/156 allot**y**pes are independent of TPN for cell surface expression, they might have very less ER retention time and might facilitate the export of HLA-A^*^24 molecules loaded with IE-1 peptides to the cell surface during HCMV infection.

To understand how residue 156 impacts on the function within this common HLA-A^*^ group, the aim of the present study was to investigate the mode of peptide recruitment and interaction of HLA-A^*^24 variants and the PLC with regard to micropolymorphism at position 156 in the HLA heavy chain.

## 2. Materials and Methods

### 2.1. Cell Lines and Lentiviral Vectors

Human B-LCL 721.220 is lacking functional TPN and both HLA-A and HLA-B genes [15] while 721.221 lacks functional HLA class I genes [30].

cDNA from a HLA-A^*^24:02 positive donor (exon 1 through exon 7) was amplified by PCR and products were cloned using TOPO-TA-Cloning Kit (Invitrogen, Karlsruhe, Germany). HLA-A^*^24:06 and HLA-A^*^24:13 variants were generated by mutating Glu at residue 156 to Trp or Leu, respectively, by SDM (QuickChange Multi-Site-Directed Mutagenesis Kit, Stratagene, Amsterdam, The Netherlands). HLA-A^*^24 inserts were subsequently cloned into pRRL.PPT.SFFV.mcs.pre vector.

Soluble HLA-A^*^24/156 variants were generated from pRRL.PPT.SFFV.mcs.pre/A^*^24 vectors by SDM to introduce a stop codon after exon 4.

To construct shRNA-expressing lentiviral vectors for silencing of TPN, stable short hairpin RNA (shRNA) expression cassettes were cloned into the pLVTHm/si vector containing eGFP as reporter [6].

Lentiviral particles were produced and B-LCLs transduction was performed as described earlier.

### 2.2. Analysis of HLA-A^*^24 mRNA Levels and A^*^24 Protein Levels

mRNA levels of HLA-A^*^24 in transduced cells or TPN in TPN-silenced cells were measured by real time PCR using StepOnePlus Real-Time PCR system (Applied Biosystems).

Surface expression of HLA-A^*^24/156 variants was assessed by flow cytometry using anti-HLA-A9-FITC (OneLambda) or w6/32-PE (Biolegend)abs. Cells were analyzed using FACS Canto A (BD Biosciences) and FLOWJO software.

sHLA producing clones were grown in bioreactors* CELLine* (Integra, Fernwald, Germany) and protein levels were determined by sandwich ELISA using w6/32 (Serotec, Duesseldorf, Germany) [31, 32] monoclonal antibody (mAb) as capture antibody. The supernatants were affinity-purified using NHS-activated* HiTrap* columns coupled to mAb w6/32.

### 2.3. Characterization of sHLA-A^*^24-Derived Peptides

Peptides from sHLA-A^*^24/156 complexes (4 mg total protein from each variant) were differentiated into low and high binding peptides. The trimeric elution fractions were filtered through a 10 kDa MWCO membrane (Millipore, Schwalbach, Germany) and peptides detected in the flow-through were considered to be low binding. The retentate containing sHLA-A^*^24 trimeric complexes was treated with 0.1% trifluoroacetic acid (TFA) to elute high binding peptides. Fractions containing low or high affinity peptides were subjected to an Eksigent Nano-LC Ultra 2D HPLC coupled to an Orbitrap ion trap (Thermo Fischer, Waltham, Massachusetts, USA). Database queries were carried out using Mascot software [33].

### 2.4. Assessment of Interactions of HLA-A^*^24 with the PLC Components

Lysates of HLA-A^*^24/156 expressing cells were immunoprecipitated with protein A-sepharose beads (GE Healthcare, Munich, Germany) covalently coupled to rabbit polyclonal TAP1 (Enzo Life Sciences) or PaSta1 (directed towards TPN kindly provided by Peter Cresswell, Yale University, New Haven, CT) abs. Where TAP1 was used for immunoprecipitation, mouse mAb to ERp57 and CRT and rabbit polyclonal TPN conjugated to HRP (Enzo Life Sciences) were used as primary abs. For immunoprecipitations with PaSta1, rabbit polyclonal to ERp57 and TAP1 and CRT (Enzo Life Sciences) were used as primary abs. Mouse anti-HLA-A mAb (Santa Cruz) was used to detect the heavy chain. Blots were incubated with respective HRP conjugated secondary abs.

### 2.5. Computation Analysis

We modeled the AA exchanges at 156 on the HLA-A^*^24:02 structure (PDB 3I6L) [29] using the Site Directed Mutator (SDM) server (http://mordred.bioc.cam.ac.uk/sdm/sdm.php) [34] and the FoldX plugin for Yasara [35]. The graphics program PyMOL (http://www.pymol.org/) was used to generate all structure figures.

## 3. Results

### 3.1. HLA-A^*^24/156 Variants Are Expressed at Varying Levels on the Surface of 721.220 Cells

**Figure 1:**
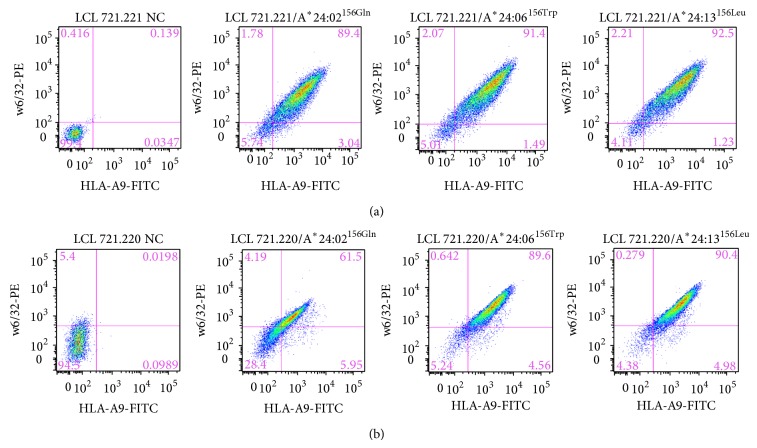
HLA-A^*^24/156 variants are expressed at different levels on the surface 721.220 (HLA-/TPN−) or 721.221 (HLA-/TPN+) cells. Flow cytometric analysis of HLA-A^*^24/220 and HLA-A^*^24/221 cells stained with anti-HLA-A9-FITC and w6/32-PE conjugated mAbs. Figures 1(a) and 1(b) show expression of HLA-A^*^24/156 variants on the surface of 721.221 and 721.220 cells, respectively. All the three allelic variants, HLA-A^*^24:02^156Gln^, HLA-A^*^24:06^156Trp^, and HLA-A^*^24:13^156Leu^, showed high levels (~92%) of expression on the surface of 721.221 cells. In 721.220 cells, HLA-A^*^24:06^156Trp^ and A^*^24:13^156Leu^ were expressed at relatively higher levels (~90%) as compared to HLA-A^*^24:02^156Gln^ (~60%).

Analysis of surface expression of HLA-A^*^24/156 variants demonstrated all the allelic variants to be expressed at high levels on the surface of HLA-A^*^24 transduced 721.221 cells (Figure 1(a)). In 721.220 cells, HLA-A^*^24:06^156Trp^ or HLA-A^*^24:13^156Leu^ were expressed at high levels while HLA-A^*^24:02^156Gln^ showed relatively low levels of surface expression (Figure 1(b)). This suggested TPN independent mode of peptide presentation for the HLA-A^*^24:06^156Trp^ and HLA-A^*^24:13^156Leu^ and partial dependence on TPN by HLA-A^*^24:02^156Gln^.

### 3.2. TPN Silencing in HLA-A^*^24/221 Cells Confirms the Relative Levels of TPN Independence Conferred by Residue 156

**Figure 2:**
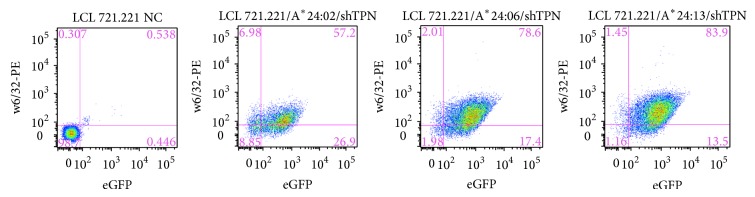
Silencing of TPN does not affect the relative surface expression of HLA-A^*^24/156 variants. Expression of HLA-A^*^24/156 variants on the surface of 721.221 cells transduced with shRNA targeting TPN. Flow cytometric analysis for GFP (reporter gene from pLVTHM/si vector) and w6/32-PE showed all the HLA-A^*^24/156 variants to be GFP positive. Both HLA-A^*^24:06 and HLA-A^*^24:13 were highly positive for w6/32 (~80%) while relatively lower percentage of A^*^24:02 was positive for w6/32 (~60%) thus confirming relative levels of TPN independence exhibited by the HLA-A^*^24/156 variants.

To confirm that the expression of HLA-A^*^24/156 variants on the surface of 721.220 cells is due to their TPN independent status, we performed reciprocal experiments by silencing TPN in HLA-A^*^24 transduced 721.221 cells. Four weeks after silencing, the surface expression levels of HLA-A^*^24 in these cells were assessed by flow cytometry using w6/32-PE conjugated ab. HLA-A^*^24:06^156Trp^ and HLA-A^*^24:13^156Leu^ were expressed on the surface of shTPN/221 cells at high levels while HLA-24:02^156Gln^ showed relatively lower levels of surface expression (Figure 2), similar to HLA-A^*^24 expression levels observed in 721.220 cells.

### 3.3. HLA-A^*^24/156 Variants Show Similar Association Levels with TAP

**Figure 3:**
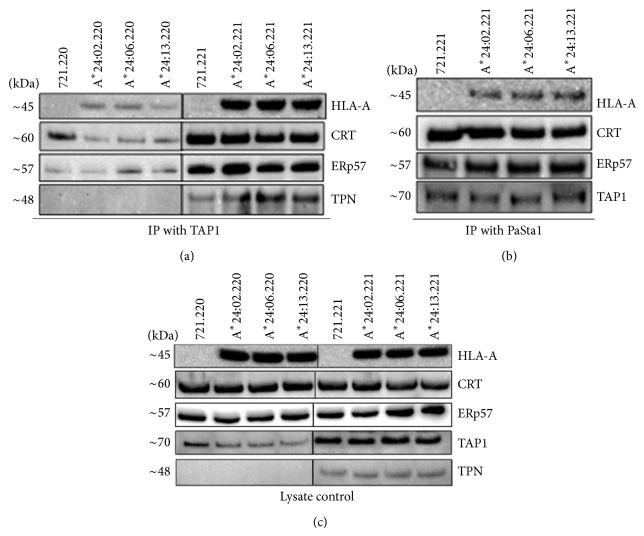
HLA-A^*^24/156 variants are associated at similar levels with TAP and TPN. Lysates from HLA-A^*^24 transduced 721.221 cells were immunoprecipitated with TAP1 (Figure 3(a)) or with PaSta-1 (anti-TPN) antibody (Figure 3(b)). The immunoprecipitates were subjected to SDS-PAGE and transferred to PVDF membranes that were probed with antibody against HLA-A heavy chain, CRT, ERp57, TPN, and TAP1. All the 3 HLA-A^*^24/156 variants showed similar levels of association with TPN in 721.221 cells indicating that they make use of TPN when it is present (Figure 3(b)). HLA-A^*^24/156 variants showed strong associations with TAP1 in 221 cells (Figure 3(a)). They were weakly associated with TAP1 in 220 cells lacking functional TPN. Lysate controls showed decreased steady state TAP1 levels in 220 cells compared to 221 cells (Figure 3(c)). Lysate controls showed similar levels of HLA-A24, CRT, and ERp57 in all the transduced 721.220 and 721.221 cells (Figure 3(c)) and similar levels of TPN in all the transduced 721.221 cells.

To assess the influence of AA polymorphisms at position 156 on the association of HLA-A^*^24 variants with TAP, HLA-A^*^24/156 transduced 721.220 or 721.221 cells were immunoprecipitated with anti-TAP1 ab. The coprecipitated members were detected by blotting with abs directed against the PLC components or HLA-A hc (Figure 3(a)). CRT, ERp57, and HLA-A^*^24 heavy chain in all the three variants showed strong associations with TAP in 721.221 cells. However, very faint bands appeared for CRT, ERp57, and heavy chain among the immunoprecipitates from transduced 721.220 cells. Lysate controls showed similar levels of CRT, ERp57, and HLA-A^*^24 in all the 721.220 and 721.221 transduced cells (Figure 3(c)). We hypothesized that the decreased association levels of HLA-A^*^24 with TAP in cells lacking TPN might be due to reduced TAP expression levels in these cells. Therefore, we compared the intracellular TAP levels in HLA-A^*^24 expressing 721.220 and 721.221 cells. Lysates from these cells were immunoblotted wih anti-TAP1 ab. We found decreased steady state TAP expression levels in 721.220 cells as compared to 721.221 cells.

### 3.4. HLA-A^*^24/156 Variants Are Associated with TPN in 721.221 Cells

Given that the HLA-A^*^24/156 variants are relatively independent of TPN for their surface expression, we analysed their association with TPN when it is present. Lysates from HLA-A^*^24 transduced 721.221 cells were immunoprecipitated with anti-TPN ab and blotted with abs specific for the individual PLC components or the HLA-A hc. All the 3 allelic variants showed similar levels of association with TPN in 721.221 cells indicating that they make use of TPN when it is present (Figure 3(b)).

### 3.5. Distinct Peptide Profiles Exhibited by HLA-A^*^24/156 Variants in the Presence and Absence of TPN

**Figure 4:**
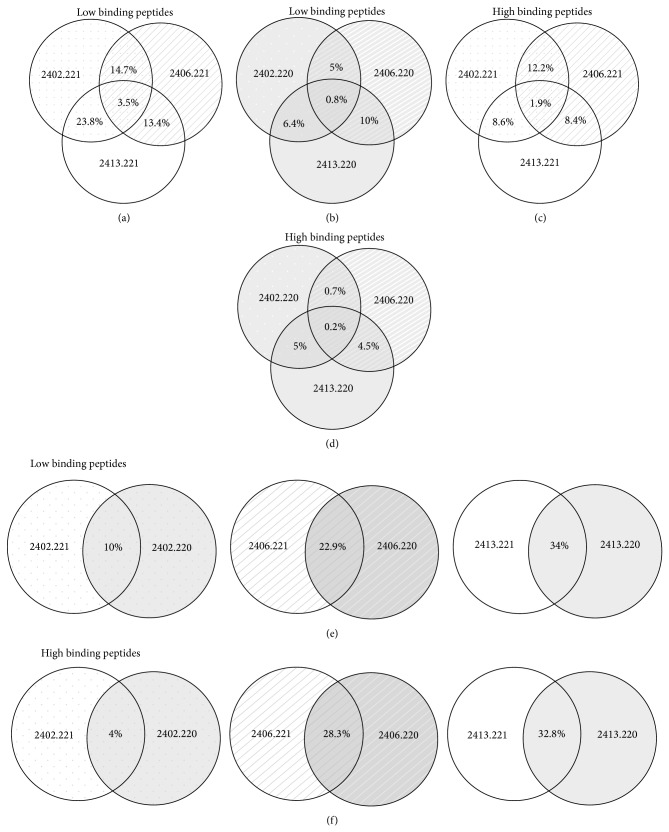
Shared peptides of HLA-A^*^24/156 allotypes. Percentages of low binding peptides shared between sHLA-A^*^24:02, sHLA-A^*^24:06, and sHLA-A^*^24:13 in the presence (a) or absence (b) of TPN. Percentage of high binding peptides shared between the 3 allotypes in the presence (c) or absence (d) of TPN. (e) and (f) demonstrate percentages of low and high binding peptides shared by individual sHLA-A^*^24/156 variants when presented in the presence and absence of TPN. sHLA-A^*^24/156 variants in the absence of TPN were found to share a very small percentage of their overall peptide repertoire compared to peptide repertoire shared by them in the presence of TPN.

Mass spectrometric analysis used in this study had a high mass accuracy in the range of 10 ppm. Soluble HLA- (sHLA-) A^*^24 molecules were expressed in 721.220 (HLA-/TPN−) or 721.221 (HLA-/TPN+) cells and their derived peptides were categorized as low or high binding peptides (see Materials and Methods) (Figure 4). Analysis of peptides presented by sHLA-A^*^24:02^156Gln^ in the presence of TPN (721.221 cells) demonstrated the majority of peptides being of low binding (~82%). In sHLA-A^*^24/156 expressing 721.221 cells, we obtained 113, 215, and 165 low binding peptides from sHLA-A^*^24:02^156Gln^, sHLA-A^*^24:06^156Trp^, or sHLA-A^*^24:13^156Leu^, respectively. Upon TFA treatment, we were able to acquire 25 high binding peptides derived from sHLA-A^*^24:02^156Gln^, 148 from sHLA-A^*^24:06^156Trp^, and 64 from sHLA-A^*^24:13^156Leu^. We found that sHLA-A^*^24:06^156Trp^ in the absence of TPN (721.220 cells) presented a significantly higher percentage of high binding peptides compared to low binding ones. From sHLA-A^*^24/220 cells, we were able to recover 42 low binding and 2 high binding peptides unique to sHLA-A^*^24:02^156Gln^, 90 low binding and 136 high binding peptides from sHLA-A^*^24:06^156Trp^, and 100 low binding and 21 high binding peptides from sHLA-A^*^24:13^156Leu^.

### 3.6. Peptide Length

**Figure 5:**
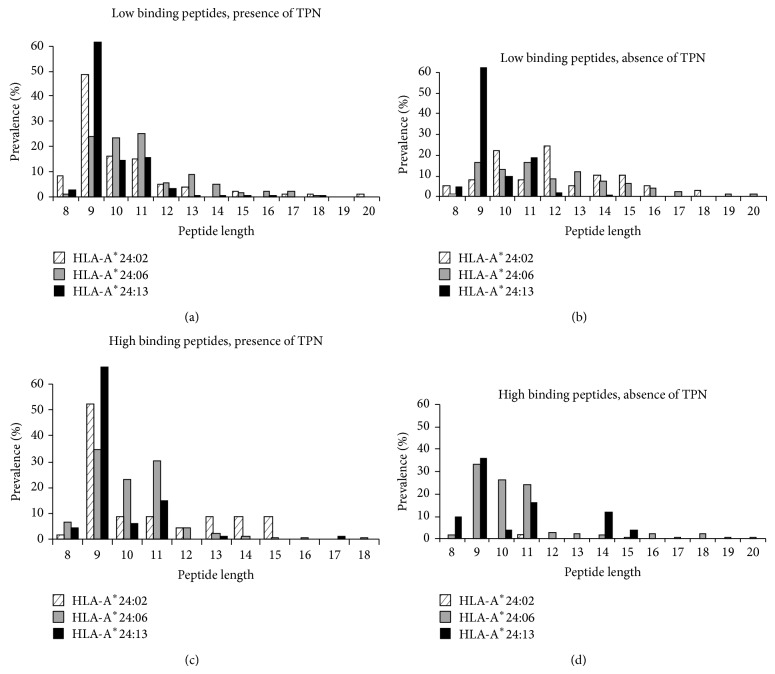
Length of peptides presented by HLA-A^*^24/156 variants. *x*-axis represents the peptide length and *y*-axis represents percentage prevalence of peptides of a given length. Crossed, grey, and black bars represent values from sHLA-A^*^24:02, sHLA-A^*^24:06, and HLA-A^*^24:13, respectively. sHLA-A^*^24/156 variants were found to present a large number of peptides of extraordinary length notably in the absence of TPN.

The majority of peptides presented by sHLA-A^*^24/156 variants in the presence of TPN were found to be of canonical length (8–10 AA) (Figure 5). Absence of TPN resulted in the presentation of a significantly higher percentage of noncanonical peptides by HLA-A^*^24:02^156Gln^ (77%) and HLA-A^*^24:06^156Trp^ (79%). We also identified peptides of extraordinary lengths (≥15 AA) from all the 3 subtypes (Figure 5).

### 3.7. Frequency of Amino Acid Residues at Peptides *p*2 and *pΩ*

Analysis of peptides presented in both the presence and the absence of TPN demonstrated Y to occur most frequently at p2 in all the HLA-A^*^24/156 variants (Figure 5). In addition, we also found significantly higher occurrence of F at p2. HLA-A^*^24:02 restricted high binding peptides are preferentially anchored by A (8%) or E (8%) at p2. Absence of TPN resulted in change in the binding preference for HLA-A^*^24:02 at p2 from Y (12.8%) to S (15.3%). In addition, they also preferred to bind K (10.2%), L (10.2%), F, I, and V (7.6%) at p2. Among low binding peptides eluted from sHLA-A^*^24:06, L was found to occur at higher frequency in A^*^24:06 (9.4%) compared to A^*^24:02 (2.7%) and A^*^24:13 (2.9%). Low binding peptides eluted from sHLA-A^*^24:06/220 demonstrated significantly higher frequency of L (16%) and A (8%) occurring at p2 compared to sHLA-A^*^24:02 (10.2%, 2.5%) and sHLA-A^*^24:13 (3%, 2%). We also found W (5%) at p2 unique to peptides derived from sHLA-A^*^24:06 in the absence of TPN. Among the high affinity peptides presented in the absence of TPN, Y (47%) and F (22% for A^*^24:06, 23.5% for A^*^24:13) occurred frequently at p2 in addition to L (9.8% for A^*^24:06 and 5% for A^*^24:13) and A (6.8% for A^*^24:06 and 11.7% for A^*^24:13).

Comparison of AA frequencies at pΩ demonstrated F or L to occur most frequently among peptides derived from sHLA-A^*^24/156 variants. Low binding peptides derived from sHLA-A^*^24:02 in the absence of TPN showed preference for D (30.7%) and A (10.2%) in addition to L (20.5%) and F (10.2%). Among high binding peptides eluted from sHLA-A^*^24:02, D (12%) and I (8%) were also found to occur more frequently. K (11.2%) at pΩ occurred more frequently among low binding peptides derived from sHLA-A^*^24:06 in both the presence and the absence of TPN.

### 3.8. Analysis of Shared Peptides

A small percentage of overall peptide repertoire was found to be shared among all the three HLA-A^*^24/156 variants both in the presence and in the absence of TPN. Among the peptides eluted from sHLA-A^*^24/221 cells, ~3.5% of low binding and 1.9% of high binding peptides were shared between all the 3 variants. In the absence of TPN, only 0.8% of low binding peptides and 0.2% of high binding peptides were shared between them. Also, the shared peptide percentage of HLA-A^*^24:02^156Gln^ in the presence and absence of TPN (shared: 10% low binding, 4% high binding) was found to be lower than the peptide repertoire shared by HLA-A^*^24:06^156Trp^ (shared: 22.9% low binding, 28.3% high binding) and HLA-A^*^24:13^156Leu^ (shared: 34% low binding, 32.8% high binding).

### 3.9. Molecular Modeling

HLA-A^*^24:02 contains the residue triad Met97/His114/Gln156 that appears to influence the stability of the PBR and dictate PLC independent peptide loading. The measure of surface expression is low for HLA-A^*^24:02^156Gln^ but high for HLA-A^*^24:06^156Trp^ and HLA-A^*^24:13^156Leu^ allotypes in the absence of TPN (721.220 cells). To investigate the structural basis of this TPN dependency in HLA-A^*^24 alleles we generated molecular models using the structure of HLA-A^*^24:02^156Gln^ as a template and mutating residue 156 to HLA-A^*^24:06^156Trp^ or HLA-A^*^24:13^156Leu^ (Figure 8). To predict the magnitude of the HLA-A^*^24:06^156Trp^ or HLA-A^*^24:13^156Leu^ polymorphisms on the protein structure we used the Site Directed Mutator (SDM) server (http://mordred.bioc.cam.ac.uk/sdm/sdm.php) [34]. The SDM server calculates a stability score that is based upon environment-dependent AA substitution and propensity tables derived from homologous protein families. The stability score (Pseudo ΔΔ*G*) is negative when a mutation is destabilizing while a positive score indicates that a particular mutation is stabilizing thereby allowing us to predict the effect that a particular mutation will have on the stability of proteins. The HLA-A^*^24:06^156Trp^ or HLA-A^*^24:13^156Leu^ polymorphisms both illustrated high stability score values of 2.37 and 3.11, respectively, suggesting that they have increased stability. To confirm the predicted increase in stability we also calculated the predicted difference in free energies between wild type and mutant proteins (ΔΔ*G* values) using FoldX [35]. Again the HLA-A^*^24:06^156Trp^ or HLA-A^*^24:13^156Leu^ polymorphisms both illustrated ΔΔ*G* values of −0.18 and −1.44, respectively, consistent with increased stability and suggesting that these mutations are likely to provide enhanced TPN independence through generating an energetically stable and a more peptide receptive environment.

## 4. Discussion

Clinical outcome of unrelated bone marrow transplantation over the last years demonstrated the value of knowledge about the impact of distinct HLA class I mismatches. The feasibility of using mismatched transplants is appreciated by the extensive datasets that have been collected over the years, including clinical data, high resolution sequencing, peptide sequencing, and T-cell studies.

Given the fact that every HLA allele has an individual peptide repertoire, it becomes obvious how many single peptide-HLA molecules are available for an antigenic T-cell. For that reason it has been proposed that whether GvHD and GvL can be related to the recipient specific peptide repertoire and its set of shared peptides by the donor HLA molecule should be assessed [36]. While the risks associated with HLA-mismatches for HSCT are clear, it is important to understand how certain peptides are selected by distinct alleles and thus how a given mismatch can influence the peptide selection and presentation process.

The conventional peptide selection and presentation is known to be driven by several proteins including TAP and TPN which import and edit peptides for being loaded and presented by the individual HLA class I molecule. A key component of the PLC is the protein TPN that orchestrates peptide selection by facilitating the stabilization of TAP and promoting the binding and translocation of peptides by TAP. The absence of TPN abrogates the binding of class I molecules to TAP and results in unstable pHLA complexes with short half-life times making it one of the main targets for viruses to interfere with the presentation of viral peptides to CTLs. Despite its dedicated function certain HLA alleles are able to circumvent the PLC and can therefore present an alternate repertoire of peptides due to their PLC independence. These TPN independent alleles are therefore advantageous in viral infections such as hepatitis C (HCV) [37] or HIV [38].

We recently described AA position 156 in the heavy chain of B^*^44 variants to influence PLC association and therefore peptide repertoire and selection [6]. To understand if position 156 might also influence PLC association in a common HLA-A^*^ group, we carefully choose A^*^24, an allelic group that is relatively common in the Caucasian population (A^*^24:02 ~20%). Since TPN independence might permit distinct alleles to present poorly tolerated self-peptides to the self-immune sytem, a probability of autoimmune reactions should be considered. In the view of this context, it becomes interesting that HLA-A^*^24:02 is found to be a predisposing factor for type 1 diabetes [39] and proliferative diabetic retinopathy [40]. TPN-independency might also permit certain HLA variants to present viral antigens during an infection. HCMV infected human fibroblasts were found to naturally process and present the immediate early-1 antigen derived peptides on HLA-A^*^24:02 [26]. Given the implication of HLA-A^*^24:02 with autoimmune disorders and its ability to present viral peptides, we wanted to investigate if polymorphisms occurring at position 156 influence PLC dependence, structure and property of the PBR, and subsequently peptide features and repertoire presented by these allotypes.

**Figure 6:**
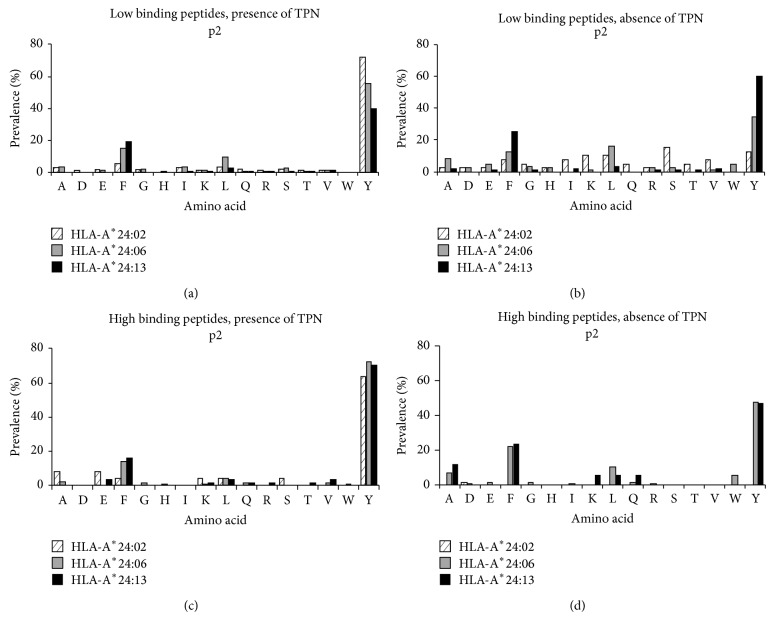
Frequency of amino acids occurring at p2 in peptides eluted from HLA-A^*^24/156 variants. *x*-axis represents AA residues at p2 occurring in sHLA-A^*^24:02 (crossed), sHLA-A^*^24:06 (grey bar), and sHLA-A^*^24:13 (black bar). *y*-axis represents percentage prevalence of individual residue at p2.

**Figure 7:**
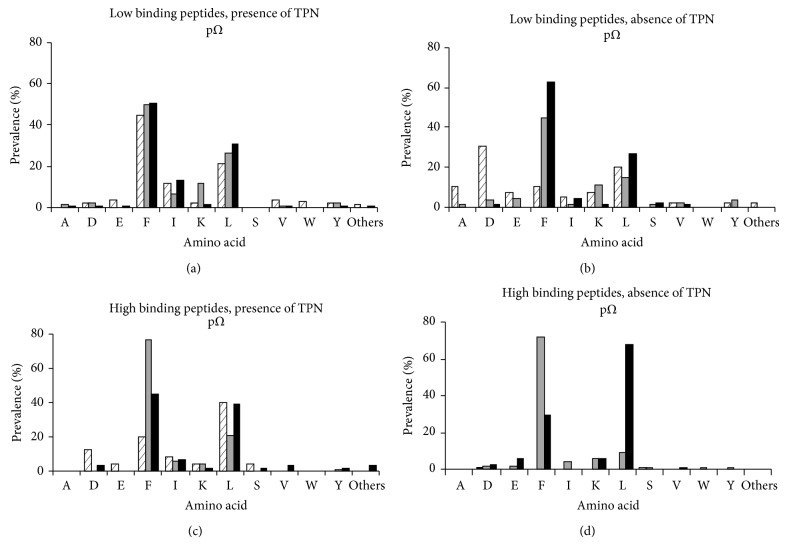
Frequency of amino acids occurring at pΩ in peptides eluted from HLA-A^*^24/156 variants. *x*-axis represents AA residues at pΩ occurring in sHLA-A^*^24:02 (crossed bar), sHLA-A^*^24:06 (grey bar), and sHLA-A^*^24:13 (black bar). *y*-axis represents percentage prevalence of individual residues at pΩ.

We systematically investigated HLA-A^*^24:02^156Gln^, HLA-A^*^24:06^156Trp^, and HLA-A^*^24:13^156Leu^. Interestingly, a difference in function for A^*^24/156 variants' PLC association and peptide selection could be observed. The peptide binding motifs at p2 and pΩ for A^*^24/156 derived peptides show in the absence of TPN high promiscuity, particularly the low binding peptides (Figures 6 and 7). Remarkably more peptides, particularly low binding peptides, of unusual length have been selected and presented in the absence of TPN (Figure 5), suggesting presentation of nonoptim**i**zed ligands with unusual length during a viral infection or downregulation of PLC components. Both A^*^24:06 and A^*^24:13 showed high levels of surface expression (~75%) in the absence of TPN, while A^*^24:02 exhibited partial independence. However no difference in association with TPN or TAP for all the allelic variants could be detected, although their peptides clearly showed different patterns and features in presence or absence of TPN. This observation demonstrates that also TPN independent alleles preferentially select and present peptides via the classical PLC pathway.

**Figure 8:**
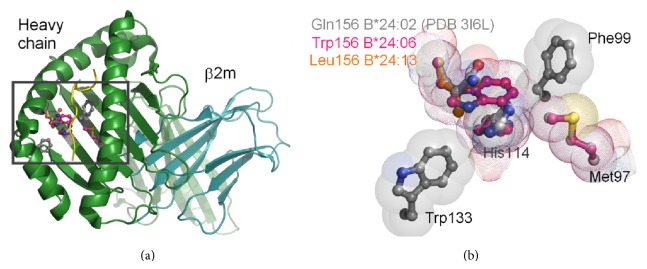
Representation of the 97/114/156 residue triad. Ribbon representation of the HLA-A^*^24:02 structure (PDB 3I6L) [29], HLA hc (green) and b2m (turquoise) and peptide (yellow); residues 97, 99, 114, 133, and 156 are drawn as ball and stick (a). A magnified view of the residues highlighted in (a); the HLA hc, b2m, and peptide have been removed for clarity (b). The residue triad Met97/His114/Gln156 from the HLA-A^*^24:02 structure is shown as ball and stick with a transparent van der Waals surface (grey). The HLA-A^*^24:06^156Trp^ (pink) or HLA-A^*^24:13^156Leu^ (orange) polymorphisms, depicted as a dotted van der Waals surface, show increased energetically stability provided by improved packing between the triad residues and aromatic residues Phe99 and Trp133.

The question remains as to what makes A^*^24:06 and A^*^24:13 more independent of TPN than A^*^24:02. To understand this, the three molecules were analyzed based on their structural features. Structurally, HLA-A^*^24:02 contains the residue triad Met97/His114/Gln156 (Figure 8), while A^*^24:06 and A^*^24:13 contain Trp and Leu polymorphism, respectively, at 156 that provides TPN independence by stabilizing the triad residues, thus generating an energetically stable and a more peptide receptive environment. Structural modeling and estimation of the free energy difference between wild-type and mutant proteins indicated that the HLA-A24/156 allotypes had increasing stability in the order of HLA-A^*^24:02^156Gln^, HLA-A^*^24:06^156Trp^, and HLA-A^*^24:13^156Leu^. We also investigated other known TPN independent alleles using the described methods. The HLA-B^*^44 group contains two independent mutations that are able to influence PLC dependence, HLA-B^*^44:02^156Asp^ > HLA-B^*^44:28^156Arg^ and HLA-B^*^44:02^116Asp^ > HLA-B^*^44:05^116Tyr^. Our analysis of these polymorphisms also demonstrated Δ*G* values indicative of increased protein stability.

The further reduction of HSCT associated risks relies on our understanding of how successful clinical outcomes can be achieved despite patient-donor allelic mismatches. The clinical importance of single mismatches at specific residues like position 156 is well known and is associated with acute Graft versus Host Disease in the B^*^44 allelic group [23] and strong* in vitro* T-cell responses in A^*^02 [41–43], B^*^35 [44], and B^*^44 [25] subtypes. The differences in both TPN dependency and peptide repertoire for variants with single mismatches at residue 156 described in the B^*^44 group [6] and here in the A^*^24 group highlight the need for systematic analysis to understand the impact of HLA mismatches. Although advantageous for viral immunity the presentation of unusual ligands by TPN independent alleles could also be considered disadvantageous and representing an overlooked factor that may need to be considered more carefully during donor selection for hematopoietic stem cell transplantation (HSCT).

## Supplementary Material

Supplementary Tables 1, 2, and 3 present HLA-A^*^24:02, A^*^24:06 or A^*^24:13 restricted self-peptides of low or high affinity acquired in the presence (source LCL 721.221 cells) or absence (source LCL 721.220 cells) of Tapasin.

## Abbreviations

HSCT: Hematopoietic stem cell transplantation
GvHD: Graft versus Host Disease
pHLA: Peptide-HLA
PLC: Peptide loading complex
PBR: Peptide binding region
ER: Endoplasmic reticulum
AA: Amino acid
TPN: Tapasin
CRT: Calreticulin
LCL: Lymphoblastoid cell line.
