# Supplementary material for: A Micropolymorphism Altering the Residue Triad 97/114/156 Determines the Relative Levels of Tapasin Independence and Distinct Peptide Profiles for HLA-A*24 Allotypes
Source: J Immunol Res. 2014 Dec 4;2014:298145. doi: 10.1155/2014/298145 (PMC4353853; doi:10.1155/2014/298145)
Supplement: Supplementary file 1 — Supplementary Tables 1, 2, and 3 present HLA-A*24:02, A*24:06 or A*24:13 restricted self-peptides of low or high affinity acquired in the presence (source LCL 721.221 cells) or absence (source LCL 721.220 cells) of Tapasin. [file 298145.f1.pdf]

Supplementary Table 1:

HLA-B*24:02 derived self peptides

origin: LCL 721.221 cells / Low binding peptides (before TFA)

Peptide position
1 2 3 4 5 6 7 8 9 10 11 12 14 15 16 17 18 19 20

**Ligands**									**Source**
R	Y	V	P	R	A	S	Y		G1/S-specific cyclin-D3
R	I	S	P	D	R	V	Y		Macrophage migration inhibitory factor
K	Y	P	S	P	F	F	V		ATP-dependent RNA helicase A
T	M	S	A	L	I	L	T		Carboxypeptidase D
I	Y	V	L	P	M	L	T		Leu-tRNA ligase, cytoplasmic
Y	A	E	K	L	H	R	L		Dystonin
Y	S	K	Q	M	Q	R	F		Histone deacetylase 2
S	Y	Q	K	V	I	E	L		Lymphokine-activated killer T-cell-originated prot. kinase
L	E	K	S	F	Y	Q	F		Superkiller viralicidic activity 2-like 2
N	Y	I	E	K	V	V	A	I	Abl interactor 1
A	Y	V	H	M	V	T	H	F	Bax inhibitor 1
T	Y	G	E	I	F	E	K	F	NADH dehydrogenase [ubiquinone] 1 subunit C2
N	Y	G	R	V	F	S	E	W	Sorting nexin-4
V	Y	G	P	L	P	Q	S	F	Cytochrome c oxidase subunit 4 isof. 1
K	Y	I	S	K	P	E	N	L	Calcium-bind. prot. 39
K	Y	A	H	M	I	N	G	F	Adenylosuccinate synthetase isozyme 2
K	Y	V	E	R	I	H	Y	I	Heme oxygenase 2
E	Y	L	K	L	L	H	S	F	Sterol regulatory element-bind. prot. 2
I	Y	I	K	H	P	H	L	F	Deoxynucleotidyltransferase terminal-interacting prot. 1
K	Y	P	L	L	I	S	R	I	Rho guanine nucleotide exchange factor 2
Y	Y	Q	D	T	P	K	Q	I	Nucleolar prot. 16
F	Y	H	K	Y	F	N	Y	L	Exostosin-2
K	Y	K	G	I	F	N	G	F	Phosphate carrier prot., mitochondrial
K	Y	I	D	Q	K	F	V	L	Ser/thr-prot. phosphatase 2A 56 kDa reg. subunit δ isof.
A	Y	H	N	S	P	A	Y	L	SWI/SNF-related matrix-associated actin-dependent regulator
F	D	L	D	Y	G	F	Q	R	Heterogeneous nucl. ribonucleoprot. C-like 1
V	Y	V	Q	N	V	V	K	L	AP-3 complex subunit δ-1
V	Y	V	S	R	P	S	H	F	Signal transducer and activator of transcription 2
S	Y	I	A	L	P	L	T	L	Nodal modulator 2
I	L	G	P	P	P	P	S	F	Matrin-3
I	Y	I	K	Q	I	K	T	F	Retinoblastoma-like prot. 2
I	Y	A	P	P	L	P	S	L	A-kinase anchor prot. 1

K	Y	I	Q	R	Q	E	T	I		Interferon-induced GTP-bind. prot. Mx1
V	Y	I	H	H	F	D	R	I		MOB kinase activator 3A
V	F	K	E	K	H	H	S	W		Ribonuclease H2 subunit B
E	Y	V	E	K	F	Y	R	I		DNA repair prot. RAD50
P	Q	P	D	V	F	P	L	F		Prot. AATF
A	Y	I	N	K	V	E	E	L		Acyl-CoA-bind. prot.
R	Y	I	A	N	T	V	E	L		Dolichyl-diphosphooligosaccharide-prot. Glycosyltr. sub. 2
K	Y	P	D	R	V	P	V	I		γ-aminobutyric acid receptor-associated prot.
H	Y	V	E	K	P	S	T	F		Mannose-1-phosphate guanyltransferase α
N	Y	V	Q	A	V	E	E	F		Nucl. autoantigenic sperm prot.
V	Y	V	V	G	T	A	H	F		TraB domain-containing prot.
V	Y	V	K	H	S	I	S	F		AP-3 complex subunit mu-1
I	Y	G	E	K	T	Y	A	F		Serpin B10
N	Y	I	G	L	I	N	R	I		Interf.-induced, double-stranded RNA-activated prot. kinase
R	Y	F	K	G	P	E	L	L		Casein kinase II subunit α
L	Y	T	E	K	F	E	E	F		α-taxilin
R	Y	Q	K	S	T	E	L	L		Histone H3.1t
T	Y	I	Q	K	I	F	R	M		Peroxisomal acyl-coenzyme A oxidase 3
N	Y	T	L	V	S	H	L	I		Methionine-tRNA ligase, cytoplasmic
V	F	P	E	K	G	Y	S	F		Nucleolysin TIAR
S	Y	L	P	I	S	P	T	F		Solute carrier family 23 member 2
N	Y	G	V	L	H	V	T	F		NACHT, LRR and PYD domains-containing prot. 11
G	Y	I	H	V	T	Q	T	F		Prot. NKG7
E	E	T	I	E	K	M	Q	E		60S ribosomal prot. L26
R	Y	V	K	K	L	G	D	F		Squalene synthase
K	F	I	D	P	I	Y	Q	V		RNA polymer. I-specific transcription initiation factor
E	Y	A	T	K	I	S	R	F		PITH domain-containing prot. 1
K	Y	I	H	S	A	N	V	L		Mitogen-activated prot. kinase 1
I	Y	K	P	V	T	D	F	F		Leu-rich PPR motif-containing prot., mitochondrial
K	Y	Q	K	G	F	S	L	W		Translocating chain-associated membrane prot. 1
K	Q	A	L	K	Y	F	N	L		Prot. sel-1 homolog 1
I	Y	P	E	L	Q	I	E	R	F	Amyloid β A4 precursor prot.-bind. fam. B member
R	Y	F	D	P	A	N	G	K	F	Elongation factor 2
I	S	L	K	Q	A	P	L	V	H	Clathrin light chain A
I	Y	I	S	T	L	K	T	E	F	Activator of 90 kDa heat shock prot. ATPase homolog 1
I	Y	A	D	N	Q	V	M	H	F	DNA polymer. ε catalytic subunit A
I	Y	D	P	N	L	A	F	L	R	1-phosphatidylinositol 4,5-bisphosphate phosphodiesterase γ-2
R	Y	S	G	V	N	Q	S	M	L	ATPase family AAA domain-containing prot. 2
L	Y	A	D	V	G	G	K	Q	F	Translocon-associated prot. subunit δ
I	Y	N	G	K	L	F	D	L	L	Kinesin-like prot. KIF2C
Y	Y	M	K	D	L	P	T	S	F	Phosphatidylinositol 4-kinase α

Y Y S H L E G A R F	Nucleolar pre-ribosomal-associated prot. 1
E Y P D R I M N T F	Tubulin β-2A chain
V Y P K K E L P F F	Suppressor of tumorigenicity 7 prot.
I Y H T G T F A Q I	Lon protease homolog, mitochondrial
T Y S P A L N K M F	Cellular tumor antigen p53
V Y P E K L A T K F	Proteasome-associated prot. ECM29 homolog
V Y N E N L V H M I	Pre-mRNA-splicing factor SPF27
L P E Y S G I T S L	Dynein heavy chain 2
F Y V P A E P K L A F	60S ribosomal prot. L7
K Y P S V K V S V F V	ATP-dependent RNA helicase DDX39A
E F G Y S N R V V D L	Glyceraldehyde-3-phosphate dehydrogenase
R Y I S P D Q L A D L	Alpha-enolase
F Y V P A E P K L A F	60S ribosomal prot. L-19
K Y M P N V K V A V F	Spliceosome RNA helicase DDX39B
K Y I T Q G Q L L Q F	Elongation of very long chain fatty acids prot. 5
R Y P T S I A S L A F	Mitotic checkpoint protein BUB3
K Y P G H L A A I T L	Actin-related prot. 5
V Y S P H V L N L T L	Dynamin-1
I F K P P D P D N T F	Modulator of apoptosis 1
S G P Y G G G G Q Y F	Heterogeneous nucl. ribonucleoprot. A1
R Y P D N L K H L Y L	Thyroid transcription factor 1-associated prot.
V Y V D L G G S H V F	Ribosome biogenesis prot. BMS1 homolog
T Y P E G L E V L H F	Centromere prot. J
I Y L P I A N V A R I	Nucl. transcription factor Y subunit β
A K A D G I V S K N F	40S ribosomal prot. S21
Y F I D S T N L K T H F	Zinc finger prot. 593
K Y L P Y N H Q H E Y F	Fatty acid desaturase 2
P G V T V K D V N Q Q E	40S ribosomal prot. S19
V D G E P L G R V S F E	Peptidyl-prolyl cis-trans isomer. A
F R D G D I L G K Y V D	10 kDa heat shock prot.
K I D D I R K P G E S E E	T-complex prot. 1 subunit ε
Y I K S T M G K P Q R L Y	60S ribosomal prot. L10a
A L T N G I Y P H K L V F	60S ribosomal prot. L6
I L E L A G N A A R D N K	Histone H2A type 2-B
Y A S G R T T G I V M D S G D	Actin, cytoplasmic 1
L E G K V L P G V D A L S N I	Phosphoglycerate kinase 1
H A V S E G T K A V T K Y T S S K	Histone H2B type 1-B
Q V T Q P T V G M N F K T P R G P V	40S ribosomal prot. S17-like
V T Y V P V T T F K N L Q T V N V D E N	60S ribosomal prot. L31

origin: LCL 721.221 cells / High binding peptides (after TFA)

**1**	**2**	**3**	**4**	**5**	**6**	**7**	**8**	**9**	**10**	**11**	**12**	**13**	**14**	**15**	**16**	
S	F	V	I	L	V	A	L									Solute carrier family 12 member 8
D	Y	G	I	V	A	D	L									Electron transfer flavoprotein subunit α, mitochondrial
Y	E	V	S	Q	L	K	D									Cytosolic non-specific dipeptidase
G	Y	S	N	R	V	V	D	L								Glyceraldehyde-3-phosphate dehydrogenase
T	Y	G	E	I	F	E	K	F								NADH dehydrogenase [ubiquinone] 1 subunit C2
R	Y	P	T	S	I	A	S	L								Mitotic checkpoint prot. BUB3
A	Y	V	H	M	V	T	H	F								Bax inhibitor 1
L	Y	D	P	V	I	S	K	L								Microspherule prot. 1
N	Y	G	R	V	F	S	E	W								Sorting nexin-4
K	Y	I	T	D	V	V	K	L								Small subunit processome component 20 homolog
N	Y	I	G	L	I	N	R	I								Interferon-induced, RNA-activated prot. kinase
S	Y	I	E	H	I	F	E	I								Astrocytic phosphoprot. PEA-15
V	Y	V	Q	N	V	V	K	L								AP-3 complex subunit δ-1
L	Y	T	E	K	F	E	E	F								Alpha-taxilin
I	Y	K	P	V	T	D	F	F								Leu-rich PPR motif-containing prot., mitochondrial
E	E	T	I	E	K	M	Q	E								60S ribosomal prot. L26
Q	S	V	A	Y	L	A	A	L	L							Neurofibromin
L	Y	L	K	V	K	G	N	V	F							60S ribosomal prot. L19
V	Y	A	A	G	K	A	E	R	T	L						Voltage-gated potassium channel subunit β-3
V	Y	V	D	L	G	G	S	H	V	F						Ribosome biogenesis prot. BMS1
I	L	E	L	A	G	N	A	A	R	D	N	K				Histone H2A type 2-A
F	Q	I	N	Q	D	E	E	E	E	E	D	E	D			60S ribosomal prot. L22
I	K	P	S	V	E	P	S	A	G	H	D	E	L			Stromal cell-derived factor 2-like prot. 1
Y	A	S	G	R	T	T	G	I	V	M	D	S	G	D		Actin, cytoplasmic 1
H	A	V	S	E	G	T	K	A	V	T	K	Y	T	S	S	Histone H2B type 1-A

origin: LCL 721.220 cells / Low binding peptides (before TFA)

**1**	**2**	**3**	**4**	**5**	**6**	**7**	**8**	**9**	**10**	**11**	**12**	**13**	**14**	**15**	**16**	**17**	**18**	**19**	
V	T	L	E	G	V	P	A												Intercellular adhesion molecule 5
I	I	G	N	S	T	V	I												Melanin-concentrating hormone receptor 1
V	D	V	E	P	K	V	K	S											Small nucl. ribonucleoprot. Sm D1
K	F	I	D	T	T	S	K	F											60S ribosomal prot. L3
V	Y	I	K	H	P	V	S	L											26S proteasome non-ATPase regulatory subunit 8
I	L	E	A	G	N	A	A	R	D										Histone H2A type 2-A
R	V	A	P	E	E	H	P	V	L										Actin, cytoplasmic 1
F	S	Q	F	G	P	I	E	R	A										Splicing factor, pro- and gln-rich
I	S	L	K	Q	A	P	L	V	H										Clathrin light chain A
L	Y	L	K	V	K	G	N	V	F										60S ribosomal prot. L19

I Y N G K L F D L L	Kinesin-like prot. KIF2C
L Y L K V K G N V F	60S ribosomal prot. L-7
V Y N E N L V H M I	Pre-mRNA-splicing factor SPF27
A A V L E Y L T A E	Histone H2A type 2-B
I L E L A G N A A R D	Histone H2A type 1-J
V L E L A G N A S K D	Histone H2A.V
F H E T L Y Q K A D D	Fructose-bisphosphate aldolase A
V S E K G T V Q Q A D E	60S ribosomal prot. L9
A L T N G I Y P H K L V	60S ribosomal prot. L6
N E F G Y S N R V V D L	Glyceraldehyde-3-phosphate dehydrogenase
F R D G D I L G K Y V D	10 kDa heat shock prot., mitochondrial
V T Y V P V T T F K N L	60S ribosomal prot. L31
I I R S M P E Q T G E K	60S ribosomal prot. L30
A Q E E P V A A P E P K	Ser/arg repetitive matrix prot. 1
P G V T V K D V N Q Q E	40S ribosomal prot. S19
F S L P I K E S E I I D	40S ribosomal prot. S2
I K S T M G K P Q R L Y	60S ribosomal prot. L10a
L S Q Q A Q L A A A E K F	Nascent polypeptide-associated complex subunit α
R F Q S S A V M A L Q E A	Probable tubulin polyglutamylase TTLL2
M I R E A D I D G D G Q V N	Calmodulin
F Q I N Q D E E E E E D E D	60S ribosomal prot. L22
E V V V T E I P K E E E K D	60 kDa heat shock prot., mitochondrial
I K P S V E P S A G H D E L	Stromal cell-derived factor 2-like prot. 1
A S T L Q S Q K A E G T G D A	nucleosome-bind. domain-containing prot. 4
F A L P V S G M D K P V F T D	Peptidyl-prolyl cis-trans isomer. NIMA-interacting 4
L V S N L N P E R V T P Q S L	Polypyrimidine tract-bind. prot. 1
V L D Q Q Q T P S R L A V T R	Leu-rich PPR motif-containing prot., mitochondrial
G F G D L K S P A G L Q V L N D	Elongation factor 1-β
I K D D E K E A E E G E D D R D	Heterogeneous nucl. ribonucleoprot. C-like 1
P K R K A E G D A K G D K A K V K D	Non-histone chromosomal prot. HMG-17
A K H A V S E G T K A V T K Y T S S K	Histone H2B type 1-B

origin: LCL 721.220 cells / High binding peptides (after TFA)

**1**	**2**	**3**	**4**	**5**	**6**	**7**	**8**	**9**	**10**	**11**	**12**	
I	L	E	L	A	G	N	A	A	R	D		Histone H2A type 1-B/E
A	Q	E	E	P	V	A	A	P	E	P	K	Ser/arg repetitive matrix prot. 1

Supplementary Table 2:

HLA-B*24:06 derived self peptides

origin: LCL 721.221 cells / Low binding peptides (before TFA)

Peptide position

1 2 3 4 5 6 7 8 9 10 11 12 13 14 15 16 17 18 19 20 21 22 23

Ligands									Source
T	A	G	A	K	L	L	F		Signal transducer and active. of transcription 2
D	Y	G	I	V	A	D	L		Electron transfer flavoprot. subunit α
V	Y	I	K	H	P	V	S	L	26S proteasome non-ATPase regulatory subunit 8
L	Y	I	V	H	P	T	M	F	Rho GTPase-activating prot. 1
K	Y	I	N	T	D	A	K	F	transient receptor channel 4-associated prot.
K	Y	F	H	P	P	A	H	L	Muscleblind-like prot. 2
L	Y	S	S	K	L	Y	R	F	BTB/POZ domain-containing prot. KCTD20
K	F	I	D	T	T	S	K	F	60S ribosomal prot. L3-like
V	L	P	I	V	I	I	T	F	Transmembrane prot. 111
I	Y	K	Q	D	L	P	S	L	Heat shock prot. 105 kDa
V	L	P	K	L	Y	V	K	L	Putative 40S ribosomal prot. S26-like 1
V	Y	G	P	L	P	Q	S	F	Cytochr. c oxidase subunit 4 isof. 1, mitoch.
N	Y	V	P	E	V	S	A	L	40S ribosomal prot. S17-like
V	Y	V	Q	H	P	I	T	F	Lipopolysaccharide-ind. tumor necrosis α factor
T	Y	Q	E	V	A	Q	K	F	Adenosine deaminase CECR1
A	F	I	K	I	K	M	A	F	Double-strand-break repair prot. rad21 homolog
R	Y	T	D	V	S	T	R	Y	Thioredoxin-related transmembrane prot. 2
I	F	V	K	I	L	Q	K	L	Nucl. pore complex prot. Nup133
P	Y	N	A	P	T	V	K	F	Ubiquitin-conjugating enzyme E2 C
P	Y	A	G	D	V	S	M	F	Plasminogen activator inhibitor 2
D	Y	S	S	I	L	Q	K	F	Formin-bind. prot. 1
K	Y	I	Q	R	Q	E	T	I	Interferon-induced GTP-bind. prot. Mx
K	L	P	V	T	I	N	K	F	AP-2 complex subunit α-1
V	Y	V	K	H	S	I	S	F	AP-3 complex subunit mu-1
K	Y	I	S	K	P	E	N	L	Calcium-bind. prot. 39
N	Y	F	L	D	P	V	T	I	Tripartite motif-containing prot.
I	F	S	N	V	R	I	N	L	Caveolin-1
N	Y	I	G	L	I	N	R	I	Interf.-ind., double-str. RNA-activ. prot. kinase
D	Y	V	E	G	L	R	V	F	Myosin light polypeptide 6
I	Y	V	G	R	V	K	S	F	Retinoblastoma-like prot. 1

D	Y	A	E	T	V	H	Q	L		Spectrin β chain, brain 1
K	Y	T	P	P	P	H	H	I		NADH dehydrogenase iron-sulfur prot. 5
E	Y	P	I	V	D	G	K	L		Hydroxymethylglutaryl-CoA synthase, mitochondrial
A	Y	S	D	V	A	K	R	L		Chloride intracellular channel prot. 4
V	Y	P	D	G	I	R	H	I		Splicing factor 3B subunit 3
V	Y	Q	H	L	F	T	R	I		AP-5 complex subunit ζ-1
V	Y	I	D	K	V	R	S	L		Lamin-B1
I	F	H	D	I	S	L	R	F		28S ribosomal prot. S5, mitochondrial
K	Y	F	H	P	P	A	H	L		Muscleblind-like prot. 2
I	F	H	D	I	S	L	R	F		28S ribosomal prot. S5, mitochondrial
R	Y	Q	D	I	I	H	S	I		Tyrosine-prot. kinase BAZ1B
I	F	V	D	E	V	F	K	I		E3 ubiquitin-prot. ligase UHRF1
L	Y	H	D	I	F	S	R	L		Nucl. factor erythroid 2-related factor 3
K	Y	P	D	R	V	P	V	I		γ-aminobutyric acid receptor-associated prot.
S	Y	I	S	R	T	N	Q	L		Prot. ELYS
V	I	H	K	I	F	S	K	F		Eukar. transl. initiation factor 3 subunit B
S	Y	S	Q	A	V	T	G	F		Mitoch. carrier homolog 2
V	Y	G	V	V	P	I	S	L		E3 ubiquitin-prot. ligase RNF19B
R	F	L	D	D	L	G	L	K		Desmoglein-2
V	F	K	T	T	L	N	E	F		Ser/thr-prot. kinase RIO3
V	Y	K	E	E	I	S	R	F		Transcription factor A, mitochondrial
Y	V	I	Q	K	F	F	E	F		Pumilio homolog 1
L	Y	H	A	I	S	N	H	L		TELO2-interacting prot. 2
T	Y	V	E	N	L	R	L	L		Nesprin-2
Q	S	V	A	Y	L	A	A	L	L	Neurofibromin
K	L	P	D	F	L	T	A	K	L	Apoptosis inhibitor 5
I	F	V	N	I	L	T	H	N	L	Nardilysin
T	F	H	D	H	A	L	M	I	I	Cytochrome c oxidase subunit 2
V	Y	F	A	E	R	V	T	S	L	Cysteine-rich prot. 1
A	L	P	V	I	I	F	T	T	F	V-type proton ATPase subunit e 2
I	Y	P	E	L	Q	I	E	R	F	Amyloid β A4 precursor prot.-bind. family B
E	Y	P	D	R	I	M	N	T	F	Tubulin β-4B chain
T	Y	N	D	F	I	N	K	E	L	DNA topoisomer. 2-β
V	F	F	E	V	S	P	V	S	F	2-oxoglutarate/iron-dep. domain-containing prot. 1
I	Y	P	V	D	L	G	D	K	F	DNA-dir. RNA polymer. I, II, III subunit RPABC3
V	L	K	P	G	M	V	V	T	F	Elongation factor 1-α 1
R	Y	F	D	P	A	N	G	K	F	Elongation factor 2
V	Y	K	D	M	P	E	T	S	F	Afadin
V	F	F	K	E	L	I	Q	E	F	Monocarboxylate transporter 4
A	Y	V	H	M	V	T	H	F	I	Bax inhibitor 1
I	A	V	Q	E	L	L	Q	L	K	Bifunctional glutamate/proline-tRNA ligase

L Y A D V G G K Q F	Translocon-associated protein subunit delta
I K Y P E N F F L L	Ser/thr-prot. phosphatase PP1-β catalytic subunit
R Y F D P A N G K F	Elongation factor 2
V Y K D M P E T S F	Afadin
I Y A D N Q V M H F	DNA polymerase ε catalytic subunit A
V Y S K D Q L Q T F	Beta-centractin
K F P S L F I N Q F	Threonylcarbamoyladenos. tRNA methylthiotransf.
R F P E E L T Q T F	Elongation factor 1-γ
R Y V F Q S E N T F	Suppressor of fused homolog
P I Y P E V V H M F	Ser/thr-prot. phosphat. 2A regulatory subunit
M L P L L S P V T F	Cdc42 effector prot. 3
E Y V Q D F L N H L	Polyadenylate-bind. prot.-interacting prot. 1
Y L P N Q L F R T F	ATP-dependent RNA helicase DDX1
L Y N P K I T S E L	Telomere-associated prot. RIF1
A F I R V V G S E F	26S protease regulatory subunit
L V V Y P W T Q R F	Hemoglobin subunit β
I Y Q D S F E Q R F	Cullin-4B
S F I T K I I S K F	Prot. TANC1
I Y K P V T D F F L	Leu-rich PPR motif-containing prot., mitoch.
A Y F N E K M S I L	Probable ATP-dependent RNA helicase DDX10
K I I E D L G V H F	Small subunit processome component 20 homolog
N F G P M K G G N F	Heterogeneous nucl. ribonucleoprot. A1-like 2
S Y V S I H S S G F	Spliceosome RNA helicase DDX39B
V F S T V V I H F L	3-hydroxy-3-methylglutaryl-coenzyme A reductase
M Y R P A Y L V T L	COMM domain-containing prot. 3
V Y K D H Q T I T I	DNA replication licensing factor MCM3
I Y H N P T A N S F	Vasodilator-stimulated phosphoprot.
L Y Q D Q I L E K F	Tubulin ε chain
V Y R K P K W N H L	Fatty acid desaturase 2
L N E K G Q D T G A	Myoferlin
S Y K E E I V H Q L	Adenylate kinase isoenzyme 6
I Y P S I N P P T L	Cell differentiation prot. RCD1 homolog
R Y T V G G L E T F	Tyr-prot. phosphatase non-receptor type 6
R L P E V E V P Q H L	Nucleobindin-1
D Y F S E R I K N Q F	Prot. FAM111B
K Y P K G A G R V A F	Cytoplasmic polyadenylation element-bind. prot. 2
I Y F P K K E A V T F	Phospholipase A-2-activating prot.
F Y V P A E P K L A F	60S ribosomal prot. L7
V Y V I E P H S M E F	Nucl. pore membrane glycoprot. 210
P Y F P H A D T I I Y	Transmembrane prot. 135
D Y V E I N I D H K F	Vigilin

I	Y	V	N	P	A	N	T	H	Q	F	DDB1- and CUL4-associated factor 8
A	Y	A	A	K	N	A	V	T	Q	F	Putat. cytochr. b-c1 complex subunit
I	Y	S	P	D	H	T	N	N	S	F	Transcription factor 4
V	F	I	H	K	D	K	G	F	G	F	Non-POU domain-containing octamer-bind. prot.
S	K	P	V	F	S	E	S	L	S	D	Mitoch. membr. translocase subunit Tim8 A
M	Y	I	K	Q	V	I	I	Q	G	F	Structural maintenance of chromosomes prot. 3
V	L	M	T	Q	Q	P	R	P	V	L	H/ACA ribonucleoprot. complex subunit 3
S	Y	P	M	V	L	G	A	Q	H	F	E3 ubiquitin-prot. ligase TRIM21
T	F	A	P	S	T	I	I	F	H	L	Dolichyl-diphosphooligosaccharide-prot. Glycosyltr.
Y	Y	P	T	E	D	V	P	R	K	L	60S ribosomal prot. L6
R	Y	I	S	P	D	Q	L	A	D	L	Alpha-enolase
S	E	M	E	V	Q	D	A	E	L	K	Proliferation-associated prot. 2G4
M	I	N	T	D	L	S	R	I	L	K	60S ribosomal prot. L4
G	I	F	M	K	P	S	E	L	L	K	Stromal cell-derived factor 2
I	Y	V	G	D	G	Y	V	V	H	L	HRAS-like suppressor 2
R	Y	P	T	S	I	A	S	L	A	F	Mitotic checkpoint prot. BUB3
T	F	P	N	I	A	S	A	T	K	F	Heme oxygenase 1
T	Y	V	P	V	T	T	F	K	N	L	60S ribosomal prot. L31
T	Y	T	D	V	T	P	R	Q	F	F	StAR-rel. lipid transfer prot. 7, mitochondrial
I	Y	V	D	Q	N	I	H	E	Q	L	Ubiquitin conjugation factor E4 A
T	Y	V	P	V	T	T	F	K	N	L	60S ribosomal prot. L31
T	Y	T	D	V	T	P	R	Q	F	F	StAR-rel. lipid transfer prot. 7, mitochondrial
N	L	P	D	E	E	I	F	Q	Q	L	COMM domain-containing prot. 8
N	Y	S	T	V	P	Q	K	Q	T	L	Zinc finger CCCH domain-containing prot. 14
K	Y	Q	E	V	T	N	N	L	E	F	Caprin-1
I	Y	S	P	D	H	S	S	N	N	F	Transcription factor E2-α
I	Y	G	E	K	F	E	D	E	N	F	Peptidyl-prolyl cis-trans isomer. D
I	Y	P	D	Q	K	E	N	T	H	F	Chitinase-3-like prot. 2
V	H	A	V	Q	A	L	W	K	Q	F	Interleukin-32
P	F	F	N	P	I	Q	T	Q	V	F	U5 small nucl. ribonucleoprot. 200 kDa helicase
R	F	P	P	Y	H	V	G	Q	T	F	DNA-bind. prot. A
L	Y	P	N	I	D	K	D	H	A	F	Signal transd. and active. of transcription 1-α/β
V	Y	K	H	I	K	E	A	G	M	F	Choline-phosphate cytidylyltransferase A
N	F	F	P	Y	G	D	A	S	K	F	Neuron-specific calcium-bind. prot. hippocalcin
K	Y	I	D	K	T	I	R	V	K	F	U6 snRNA-associated Sm-like prot. LSm7
S	L	P	S	A	S	H	F	S	Q	L	Prot. PRRC2C
R	F	V	N	V	V	P	T	F	G	K	40S ribosomal prot. S30
D	Y	K	D	N	F	G	S	S	S	F	Cyclin-dependent kinase 13
V	Y	R	D	G	T	G	V	V	E	F	Ser/arg-rich splicing factor 1
I	F	K	P	P	D	P	D	N	T	F	Modulator of apoptosis 1
P	Y	A	P	S	G	E	I	P	K	F	UDP-glucose 6-dehydrogenase
V	F	P	S	M	P	I	K	L	Q	L	NMDA receptor-regulated prot. 2

D Y I D P A I M K K L	Nucl. GTP-bind. prot. 1
S Y F P E I T H I V I	Tyrosine-prot. kinase JAK1
K Y M P S V K V S V F	ATP-dependent RNA helicase DDX39A
G Y A G E L G F R A F	Ubiquitin fusion degradation prot. 1 homolog
F A S E N D L P E W K	Ran-specific GTPase-activating prot.
I Y N S I N G A I T Q F	Zinc finger prot. RFP
V Y A I R E A A T N N L	Ser/thr-prot. phosphat. 2A regul. A β isof.
G F G D S R G G G G N F	Heterogeneous nucl. ribonucleoprot. A2/B1
I Y G E K F E D E N F I	Peptidyl-prolyl cis-trans isomer. A
S L V S K G T L V Q T K	Histone H1.5
A R V I T E E E K N F K	60S ribosomal prot. L13
G F V K V V K N K A Y F	60S ribosomal prot. L5
V Y G D V Q R V K I L F	Polypyrimidine tract-bind. prot. 1
I Y I D S N N N P E R F	Clathrin hc 1
R L P D G T S L T Q T F	UBX domain-containing prot. 1
Y F I D S T N L K T H F	Zinc finger prot. 593
P Y A A S P A T A A S F	RNA-bind. prot. 38
R L A K A D G I V S K N F	40S ribosomal prot. S21
V Y T P V G K A E Q G K F	Puromycin-sensitive aminopeptidase-like prot.
P Y A N Q P T V R I T E L	DNA-directed RNA polymerase II subunit RPB3
I L E L A G N A A R D N K	Histone H2A type 1
M V V E S A Y E V I K L K	L-lactate dehydrogenase B chain
V F V S E G K I L T T G F	Coronin-1A
E Q I S D I D D A V R K L	RNA polymer. II transcriptional coactivator p15
I Y T G H I Y G E E G S F	Disintegrin and metalloproteinase prot. 10
V L E L A G N A S K D L K	Histone H2A.V
Y I K S T M G K P Q R L Y	60S ribosomal prot. L10a
I L E L A G N A A R D N K	Histone H2A type 1-J
R Y P F H D S D P S A L F	Tribbles homolog 1
N Y P E Q K V V T V G Q F	Vacuolar prot. sorting-associated prot. 29
D Y A S A F Q G H D V G F	Oxidoreductase HTATIP2
V G V P R S I A A N M T F	DNA-directed RNA polymerase II subunit RPB1
A L G D P K A P G L G A F	DNA-dependent protein kinase catalytic subunit
I K P S V E P S A G H D E L	Stromal cell-derived factor 2-like prot. 1
Q G S V Q K V Y N G L Q G Y	DnaJ homolog subfamily B member 11
A S I K K G E D F V K T L K	Malate dehydrogenase, mitochondrial
T Y F P H F D L S H G S A Q V	Hemoglobin subunit α
Y A S G R T T G I V M D S G D	Actin, cytoplasmic 1
I V K T L E D I D L G P T E K	Citrate lyase subunit β-like prot., mitochondrial
I E D L S Q Q A Q L A A A E K	Nascent polypeptide-associated complex subunit α
L Q A L D F F G N P P V N Y K	Gamma-interf.-inducible lysosomal thiol reductase

L	A	G	E	E	L	A	G	E	E	A	P	Q	E	K									Hepatoma-derived growth factor-related prot. 2
H	G	S	Y	E	D	A	V	H	S	G	A	L	N	D									T-complex prot. 1 subunit α
L	E	G	K	V	L	P	G	V	D	A	L	S	N	I									Phosphoglycerate kinase 1
A	G	N	L	G	G	G	V	V	T	I	E	R	S	K									60S ribosomal prot. L22
S	S	K	Q	A	K	D	V	S	R	M	R	S	V	L									Clathrin light chain A
V	L	L	P	K	K	T	E	S	H	H	K	A	K	G									Histone H2A type 2-A
A	Q	L	G	G	P	E	A	A	K	S	D	E	T	A	A								Heat shock prot. β-1
I	Y	K	G	F	E	S	P	S	D	N	S	S	A	M	L								Actin-related prot. 2/3 complex subunit 5
L	F	V	G	N	L	P	A	D	I	T	E	D	E	F	K								Splicing factor, pro- and gln-rich
T	S	A	S	F	V	N	K	Q	P	T	L	Q	A	A	E	L							TATA-bind. prot.-associated factor 172
H	A	V	S	E	G	T	K	A	V	T	K	Y	T	S	A	K							Histone H2B type 1-J
V	G	D	V	G	Q	T	V	D	D	P	Y	A	T	F	V	K							Cofilin-1
V	Y	I	P	V	G	G	S	Q	H	G	L	L	G	T	L	F							Prot.-cys N-palmitoyltransferase HHAT
A	S	M	H	P	V	T	A	M	L	V	G	K	D	L	K	V	D						Leukotriene A-4 hydrolase
M	V	V	N	E	G	S	D	G	G	Q	S	V	Y	H	V	H	L						His triad nucleotide-bind. prot. 1
A	A	N	D	A	G	Y	F	N	D	E	M	A	P	I	E	V	K						3-ketoacyl-CoA thiolase, mitochondrial
D	G	R	G	A	L	Q	N	I	I	P	A	S	T	G	A	A	K						Glyceraldehyde-3-phosphate dehydrogenase
A	S	S	E	G	G	T	A	A	G	A	G	L	D	S	L	H	K						Actin-related prot. 2/3 complex subunit 1B
M	A	G	S	S	R	K	E	A	E	S	S	P	F	V	E	R	L	L					Endoplasmin
A	I	V	E	A	L	N	G	K	E	V	A	A	Q	V	K	A	P	L	V	L	K	D	Prot. DJ-1

origin: LCL 721.221 cells / High binding peptides (after TFA)

**1**	**2**	**3**	**4**	**5**	**6**	**7**	**8**	**9**	**10**	**11**	**12**	**13**	**14**	**15**	**16**	**17**	**18**	
P	V	T	T	F	K	N	L											60S ribosomal prot. L31
V	Y	I	K	H	P	V	S	L										26S proteasome non-ATPase regulatory subunit 8
A	Y	V	H	M	V	T	H	F										Bax inhibitor 1
K	F	I	D	T	T	S	K	F										60S ribosomal prot. L3
V	L	P	K	L	Y	V	K	L										Putative 40S ribosomal prot. S26-like 1
V	A	A	G	S	Y	Q	R	F										Polyamine-modulated factor 1
P	F	V	D	H	V	F	T	F										Ribosome biogenesis prot. BRX1 homolog
T	Y	G	E	I	F	E	K	F										NADH dehydrogenase [ubiquinone] 1 subunit C2
R	Y	P	T	S	I	A	S	L										Mitotic checkpoint prot. BUB3
V	Y	G	P	L	P	Q	S	F										Cytochrome c oxidase subunit 4 isof. 1, mitochondrial
S	Y	M	G	H	F	D	L	L										Splicing factor 3B subunit 5
N	Y	G	R	V	F	S	E	W										Sorting nexin-4
I	F	T	P	I	V	E	H	L										RNA-bind. prot. PNO1
S	F	S	T	V	H	E	K	F										WD repeat-containing prot. 36
D	Y	A	E	T	V	H	Q	L										Spectrin β chain, brain 1
N	Y	F	L	D	P	V	T	I										Tripartite motif-containing prot.
V	L	P	A	F	T	S	N	L										Poly [ADP-ribose] polymerase 4

I	Y	K	Q	D	L	P	S	L		Heat shock prot. 105 kDa
S	Q	M	K	S	Y	Q	S	F		General transcription factor 3C polypeptide 1
I	Y	T	S	S	V	N	R	L		Coatomer subunit β
P	Y	A	G	D	V	S	M	F		Plasminogen activator inhibitor 2
D	Y	G	I	V	A	D	L	F		Electron transfer flavoprotein subunit α, mitochondrial
V	Y	I	D	K	V	R	S	L		Lamin-B1
K	Y	A	H	M	I	N	G	F		Adenylosuccinate synthetase isozyme 2
D	Y	A	E	V	S	N	T	F		DNA-directed RNA polymerase III subunit RPC3
K	F	T	P	V	A	S	K	F		Zyxin
L	Y	T	E	K	F	E	E	F		Alpha-taxilin
P	Q	P	D	V	F	P	L	F		Prot. AATF
V	Y	V	V	G	T	A	H	F		TraB domain-containing prot.
Y	Y	F	P	V	K	N	V	I		Splicing factor 3B subunit 3
T	Y	Q	D	I	Q	N	T	I		DNA-directed RNA polymerase II subunit RPB1
I	Y	I	K	Q	I	K	T	F		Retinoblastoma-like prot. 2
V	Y	I	K	H	P	V	S	L		26S proteasome non-ATPase regulatory subunit 8
D	Y	M	K	T	T	S	N	F		Nucl. export mediator factor NEMF
D	Y	V	E	G	L	R	V	F		Myosin light polypeptide 6
K	Y	P	E	N	F	F	L	L		Ser/thr-pro. phosphatase PP1-β catalytic subunit
S	Y	I	E	H	I	F	E	I		Astrocytic phosphoprot. PEA-15
N	Y	V	Q	A	V	E	E	F		Nucl. autoantigenic sperm prot.
H	Y	V	E	K	P	S	T	F		Mannose-1-phosphate guanyltransferase α
V	F	P	A	I	A	Q	E	I		Cell division control prot. 6 homolog
A	Y	V	E	L	V	H	H	I		CDK5 regulatory subunit-associated prot. 1
K	Y	T	P	P	P	H	H	I		NADH dehydrogenase [ubiquinone] iron-sulfur prot. 5
I	Y	G	E	K	T	Y	A	F		Serpin B10
K	Y	P	D	R	V	P	V	I		γ-aminobutyric acid receptor-associated prot.
P	Y	V	N	N	V	P	H	L		Methionine--tRNA ligase, cytoplasmic
S	Y	A	Q	Y	V	H	N	L		39S ribosomal prot. L48
E	Y	S	K	Q	M	Q	R	F		Histone deacetylase 1
A	Y	S	D	V	A	K	R	L		Chloride intracellular channel prot. 4
E	Y	V	V	V	P	S	T	F		Calpain-1 catalytic subunit
K	Y	V	S	I	N	S	T	L		Prot. FAM208B
I	Y	K	P	V	T	D	F	F		Leu-rich PPR motif-containing prot., mitochondrial
D	Y	S	S	I	L	Q	K	F		Formin-bind. prot. 1
E	Y	Y	D	K	H	F	T	E	F	V-type proton ATPase catalytic subunit A
I	Y	S	T	V	D	D	D	H	F	Replication prot. A 32 kDa subunit
I	Y	Y	T	G	K	Y	Q	S	L	Probable E3 ubiquitin-prot. ligase MYCBP2
V	Y	A	H	I	Y	H	Q	H	F	MOB kinase activator 1A
R	Y	F	D	P	A	N	G	K	F	Elongation factor 2
A	Y	F	P	E	L	I	A	N	F	Solute carrier family 25 member 46

A	F	I	R	V	V	G	S	E	F		26S protease regulatory subunit 6B
Y	Y	S	H	L	E	G	A	R	F		Nucl. pre-ribosomal-associated prot. 1
S	F	A	K	I	Y	A	D	T	F		116 kDa U5 small nucl. ribonucleoprot. component
T	Y	T	D	V	T	P	R	Q	F		StAR-related lipid transfer prot. 7, mitochondrial
L	Y	A	D	V	G	G	K	Q	F		Translocon-associated prot. subunit δ
R	Y	P	P	K	S	G	N	Y	F		E3 ubiquitin-prot. ligase MGRN1
I	Y	P	V	D	L	G	D	K	F		DNA-directed RNA polymerases I, II, and III subunit RPABC3
E	Y	P	D	R	I	M	N	T	F		Tubulin beta-2A chain
I	Y	I	S	T	L	K	T	E	F		Activator of 90 kDa heat shock prot. ATPase homolog 1
N	Y	V	N	K	I	K	N	R	F		Paired amphipathic helix prot. Sin3a
E	Y	Y	D	K	A	F	D	R	I		Eukaryotic translation initiation factor 3 subunit D
K	Y	V	L	G	Y	K	Q	T	L		60S ribosomal prot. L30
P	Y	T	D	V	N	I	V	T	I		Isocitrate dehydrogenase [NAD] subunit α, mitochondrial
R	F	P	E	E	L	T	Q	T	F		Elongation factor 1-γ
A	Y	V	K	G	G	L	S	T	F		Exocyst complex component 2
I	Y	K	D	L	P	F	E	T	L		39S ribosomal prot. L39, mitochondrial
R	Y	F	D	G	N	L	E	K	L		E3 ubiquitin-prot. ligase
P	Y	A	K	P	I	P	A	Q	F		pre-mRNA 3' end processing prot. WDR33
H	Y	Y	R	G	N	Y	M	T	F		ATP-bind. cassette sub-family F member 1
E	Y	I	A	A	F	L	S	H	F		BRCA1-A complex subunit BRE
R	Y	Y	P	A	K	I	E	A	I		PHD finger prot. 20-like prot. 1
I	Y	P	E	L	Q	I	E	R	F		Amyloid β A4 precursor prot.-bind. family B member 1
V	F	V	G	N	I	D	D	H	F		Digestive organ expansion factor
V	Y	V	P	K	E	E	Q	L	F		Torsin-3a
Q	F	I	E	P	F	E	E	K	F		CCR4-NOT transcription complex subunit 10
K	Y	Y	E	D	N	K	Q	F	F		NADH dehydrogenase [ubiquinone] 1 α subcomplex subunit 12
V	F	P	L	L	V	A	E	T	F		Deoxyhypusine synthase
Y	Y	M	K	D	L	P	T	S	F		Phosphatidylinositol 4-kinase α
F	Y	Y	D	G	K	V	M	K	L		DNA topoisomer. 1
Q	Y	V	E	D	G	I	G	H	E	F	80 kDa MCM3-associated prot.
E	Y	A	K	P	T	R	L	N	V	F	Heterogeneous nuclear ribonucleoprot. L
E	Y	F	R	V	P	D	S	A	T	F	Vacuolar prot. sorting-associated prot.
S	Y	A	K	L	L	G	H	Q	N	L	Hyaluronan mediated motility receptor
D	Y	F	S	E	R	I	K	N	Q	F	Prot. FAM111B
N	Y	V	N	S	I	I	V	H	K	F	Prot. CLEC16A
R	Y	P	D	N	L	K	H	L	Y	L	Thyroid transcription factor 1-associated prot. 26
K	Y	I	T	Q	G	Q	L	L	Q	F	Elongation of very long chain fatty acids prot. 5
R	V	A	P	V	P	L	Y	N	S	F	Kynureninase
V	F	I	N	K	G	K	G	F	G	F	Splicing factor, pro- and gln-rich
I	F	K	P	P	D	P	D	N	T	F	Modulator of apoptosis 1
R	N	P	P	G	F	A	F	V	E	F	Ser/arg-rich splicing factor 3

T	F	P	N	I	A	S	A	T	K	F			Heme oxygenase 1
M	Y	I	K	Q	V	I	I	Q	G	F			Structural maintenance of chromosomes prot. 3
K	Y	P	D	E	N	G	F	D	A	F			Nardilysin
V	Y	V	I	E	P	H	S	M	E	F			Nucl. pore membrane glycoprot. 210
I	Y	S	P	D	H	T	N	N	S	F			Transcription factor 4
K	Y	I	D	K	T	I	R	V	K	F			U6 snRNA-associated Sm-like prot. LSm7
E	Y	V	E	A	V	S	F	Q	H	F			Translin-associated prot. X
R	F	P	P	Y	H	V	G	Q	T	F			DNA-bind. prot. A
G	Y	A	D	K	N	L	I	A	K	W			Prot. BUD31 homolog
V	H	A	V	Q	A	L	W	K	Q	F			Interleukin-32
S	Y	P	M	V	L	G	A	Q	H	F			E3 ubiquitin-prot. ligase TRIM21
L	Y	P	N	I	D	K	D	H	A	F			Signal transducer and activator of transcription 1-α/β
R	Y	I	S	P	D	Q	L	A	D	L			Alpha-enolase
T	F	A	N	I	K	L	F	N	K	F			Iron-responsive element-bind. prot. 2
N	L	P	D	E	E	I	F	Q	Q	L			COMM domain-containing prot. 8
V	F	V	P	R	D	V	P	E	S	F			WD repeat-containing prot. 75
I	Y	V	G	D	G	Y	V	V	H	L			HRAS-like suppressor 2
H	Y	V	D	L	K	D	R	P	F	F			Nucleoside diphosphate kinase A
V	Y	V	D	L	G	G	S	H	V	F			Ribosome biogenesis prot. BMS1 homolog
I	Y	V	N	P	A	N	T	H	Q	F			DDB1- and CUL4-associated factor 8
Q	Y	Q	D	T	K	G	F	S	T	W			Moesin
R	Y	A	P	G	D	A	V	E	K	W			N-acetyltransferase 10
T	Y	A	A	L	N	S	K	A	T	F			Ras GTPase-activating-like prot. IQGAP1
I	Y	K	D	D	I	S	N	S	S	F			Ras-related GTP-bind. prot. C
L	Y	Y	D	E	K	K	M	A	N	F			Hypoxia-inducible factor 1-α inhibitor
R	W	P	K	K	S	A	E	F	L	L			60S ribosomal prot. L17
R	Y	V	E	Q	K	A	G	I	T	F			ATP-dependent RNA helicase DDX50
K	Y	M	P	N	V	K	V	A	V	F			Spliceosome RNA helicase
W	Y	V	H	P	Q	V	L	Q	S	F			Chromatin assembly factor 1 subunit A
V	F	I	H	K	D	K	G	F	G	F			Non-POU domain-containing octamer-bind. prot.
T	F	A	P	S	T	I	I	F	H	L			Dolichyl-diphosphooligosaccharide-prot. glycosyltransferase
K	Y	M	P	S	V	K	V	S	V	F			ATP-dependent RNA helicase DDX39A
I	Y	I	D	S	N	N	N	P	E	R	F		Clathrin hc 1
S	L	V	S	K	G	T	L	V	Q	T	K		Histone H1.5
Y	F	I	D	S	T	N	L	K	T	H	F		Zinc finger prot. 593
A	Y	V	S	D	L	G	K	V	F	S	F		E3 ISG15—prot. ligase HERC5
A	L	S	T	G	E	K	G	F	G	Y	K		Peptidyl-prolyl cis-trans isomer. A
I	Y	N	S	I	N	G	A	I	T	Q	F		Zinc finger prot. RFP
A	Y	A	D	T	K	L	A	N	V	L	F		Dehydrogenase/reductase SDR family member 13
D	Y	A	S	A	F	Q	G	H	D	V	G	F	Oxidoreductase HTATIP2
I	L	E	L	A	G	N	A	A	R	D	N	K	Core histone macro-H2A.1
V	L	E	L	A	G	N	A	S	K	D	L	K	Histone H2A.Z

V	Y	F	K	P	S	L	T	P	S	G	E	F						Nucl. pore complex prot. Nup153
I	K	P	S	V	E	P	S	A	G	H	D	E	L					Stromal cell-derived factor 2-like prot. 1
Q	G	S	V	Q	K	V	Y	N	G	L	Q	G	Y					DnaJ homolog subfamily B member
Q	A	Q	N	T	V	T	L	A	T	S	I	K	E	K				Citrate lyase subunit β-like prot., mitochondrial
S	A	S	S	G	A	E	G	D	V	S	S	E	R	E	P			D-tyrosyl-tRNA(Tyr) deacylase 1
D	G	R	G	A	L	Q	N	I	I	P	A	S	T	G	A	A	K	Glyceraldehyde-3-phosphate dehydrogenase

origin: LCL 721.220 cells / Low binding peptides (before TFA)

**1**	**2**	**3**	**4**	**5**	**6**	**7**	**8**	**9**	**10**	**11**	**12**	**13**	**14**	**15**	**16**	**17**	**18**	**19**	**20**	**21**	**22**	
P	F	F	S	I	P	V	V															ORM1-like prot. 2
K	F	I	D	T	T	S	K	F														60S ribosomal prot. L3-like
E	E	T	I	E	K	M	Q	E														60S ribosomal prot. L26
R	Y	P	T	S	I	A	S	L														Mitotic checkpoint prot. BUB3
E	Y	P	I	V	D	G	K	L														Hydroxymethylglutaryl-CoA synthase
Y	Y	T	R	L	G	N	D	F														40S ribosomal prot. S17-like
V	Y	I	D	K	V	R	S	L														Lamin-B1
Y	Y	G	Y	D	Y	H	N	Y														Heterogeneous nucl. ribonucleoprot. Q
E	Y	S	K	Q	M	Q	R	F														Histone deacetylase 1
D	Y	S	S	I	L	Q	K	F														Formin-binding prot. 1
P	Y	N	A	P	T	V	K	F														Ubiquitin-conjugating enzyme E2 C
P	F	V	D	H	V	F	T	F														Ribosome biogenesis prot. BRX1 homolog
E	E	L	I	E	K	M	Q	E														60S ribosomal prot. L26-like 1
S	F	S	T	V	H	E	K	F														WD repeat-containing prot. 36
R	W	P	K	K	S	A	E	F														60S ribosomal prot. L17
V	W	A	A	V	P	G	K	T	F													Profilin-1
I	W	I	L	I	D	K	T	S	F													Leukocyte antigen CD37
I	Y	H	N	P	T	A	N	S	F													Vasodilator-stimulated phosphoprot.
N	Y	G	P	M	K	G	G	S	F													Heterogeneous nuclear ribonucleoprot. A3
Y	L	P	N	Q	L	F	R	T	F													ATP-dependent RNA helicase DDX1
I	Y	P	V	D	L	G	D	K	F													DNA-dir. RNA pol. I,II,III, subunit RPABC3
V	L	K	P	G	M	V	V	T	F													Elongation factor 1-α 1
V	Y	K	E	N	L	V	D	G	F													Negative elongation factor E
L	Y	A	D	V	G	G	K	Q	F													Translocon-associated prot. subunit δ
K	Y	V	L	G	Y	K	Q	T	L													60S ribosomal prot. L30
T	Y	T	D	V	T	P	R	Q	F													StAR-related lipid transfer prot. 7
I	Y	I	S	T	L	K	T	E	F													Activ. of 90 kDa HSP, ATPase homolog 1
I	Y	F	P	K	K	E	A	V	T	F												Phospholipase A-2-activating prot.
V	Y	S	Q	I	P	A	A	V	K	L												E3 ubiquitin-prot. ligase UBR5
R	F	P	P	Y	H	V	G	Q	T	F												DNA-bind. prot. A
M	Y	I	K	Q	V	I	I	Q	G	F												Struct. maintenance of chromosomes prot. 3

S K P V F S E S L S D	Mitoch. membr. translocase subunit Tim8 A
T Y V P V T T F K N L	60S ribosomal prot. L31
R Y I S P D Q L A D L	Alpha-enolase
Y Y P T E D V P R K L	60S ribosomal prot. L6
V F I H K D K G F G F	Non-POU domain-cont. octamer-bind. prot.
S N F G P M K G G N F	Heterogen. nucl. ribonucleoprot. A1-like 2
L Y P N I D K D H A F	Signal transd. active. of transcr. 1-α/β
V L M T Q Q P R P V L	H/ACA ribonucleoprot. complex subunit 3
V H A V Q A L W K Q F	Interleukin-32
F Y V P A E P K L A F	60S ribosomal prot. L7
R L P K S P P Y T A F	Eukar. translation initiation factor 4B
D K F K Y D D A E R R F	Elongation factor 1-δ
Y F I D S T N L K T H F	Zinc finger prot. 593
R L P D G T S L T Q T F	UBX domain-containing prot. 1
S L V S K G T L V Q T K	Histone H1.2
G F G D S R G G G G N F	Heterogeneous nuclear ribonucleoprot. A2/B1
A R V I T E E E K N F K	60S ribosomal prot. L13
N A V K Y L Q S L E R S	C-C motif chemokine 17
A H A E D S V M D H H F	Plasminogen activ. inhib. 1 RNA-bind. prot.
K L P K Q P V I V K A K F	60S ribosomal prot. L27a
I Y T G H I Y G E E G S F	Disintegrin, metalloprot. domain prot. 10
I L E L A G N A A R D N K	Histone H2A type 1-B/E
T Y F P H F D L S H G S A	Hemoglobin subunit α
P Y Q Y P A L T P E Q K K	Fructose-bisphosphate aldolase A
I Y I D S N N N P E R F L	Clathrin hc 1
V F V S E G K I L T T G F	Coronin-1A
G F V K V V K N K A Y F K	60S ribosomal prot. L5
V L E L A G N A S K D L K	Histone H2A.V
V Y F K P S L T P S G E F	Nuclear pore complex prot. Nup153
F A D K V P K T A E N F R	Peptidyl-prolyl cis-trans isomer. A
V L K Q V H P D T G I S S K	Histone H2B type F-S
Q G S V Q K V Y N G L Q G Y	DnaJ homolog subfamily B member 11
K E E I I K T L S K E E E T	60S ribosomal prot. L19
M V A K E A R N V T M E T E	Signal recognition particle 9 kDa prot.
D Y S G Y Q R D G Y Q Q N F	Caprin-1
F E E E A K N P G L E T H R	Aprataxin
V N V T F E G S N G T P L I	Capsid polyprot.
P D R I M N T F S V V P S P K	Tubulin beta-3 chain
S D G V I K V F N D M K V R K	Cofilin-1
P G V T V K D V N Q Q E F V R	40S ribosomal prot. S19
Q A Q N T V T L A T S I K E K	Citrate lyase subunit β-like prot.

H	G	S	Y	E	D	A	V	H	S	G	A	L	N	D								T-complex prot. 1 subunit α
L	A	G	E	E	L	A	G	E	E	A	P	Q	E	K								Hepatoma-der. growth factor-related prot. 2
R	G	G	C	G	V	V	G	G	G	G	S	C	S	S	V							Zinc finger prot. 609
A	S	N	E	D	V	T	K	A	V	D	I	T	T	P	K							PC4 and SFRS1-interacting prot.
S	Y	V	G	D	E	A	Q	S	K	R	G	I	L	T	L							Actin, cytoplasmic 1
L	F	V	G	N	L	P	A	D	I	T	E	D	E	F	K							Splicing factor, pro- and gln-rich
S	A	S	E	H	S	S	S	A	E	S	E	R	S	Y	K							Pre-mRNA-processing factor 40 homolog A
A	W	V	E	S	R	E	K	Q	A	K	G	D	T	E	F							Calcyclin-bind. prot.
S	M	G	S	Q	E	D	D	S	G	N	K	P	S	S	Y	S						COP9 signalosome complex subunit 3
H	A	V	S	E	G	T	K	A	V	T	K	Y	T	S	S	K						Histone H2B type 1-C
P	P	A	E	N	S	S	A	P	E	A	E	Q	G	G	A	E						Nuclease-sensitive element-bind. prot. 1
A	Y	V	R	L	A	P	D	Y	D	A	L	D	V	A	N	K						60S ribosomal prot. L23a
D	G	R	G	A	L	Q	N	I	I	P	A	S	T	G	A	A	K					Glyceraldehyde-3-phosphate dehydrogenase
A	S	S	E	G	G	T	A	A	G	A	G	L	D	S	L	H	K					Actin-related prot. 2/3 complex subunit 1B
A	Y	V	D	L	E	K	D	F	A	A	E	V	V	H	P	G	D	L	K			Tyrosine--tRNA ligase, cytoplasmic
K	A	S	K	T	A	E	N	A	T	S	G	E	T	L	E	E	N	E	A	G	D	Proliferation-associated prot. 2G4

origin: LCL 721.220 cells / High binding peptides (after TFA)

1	2	3	4	5	6	7	8	9	10	11	12	13	14	15	16	17	18	19	20	
H	L	T	D	A	Y	F	K													60S ribosomal prot. L6
K	Y	G	P	V	F	S	F													Lanosterol 14-alpha demethylase
L	Y	W	S	H	P	R	K	F												40S ribosomal prot. S29
V	Y	A	Q	V	A	R	L	F												Paired amphipathic helix prot. Sin3a
T	F	S	D	V	E	A	H	F												Zinc finger prot. 280C
V	A	A	G	S	Y	Q	R	F												Polyamine-modulated factor 1
L	Y	S	E	V	D	V	H	F												Pogo transposable element with ZNF domain
I	F	A	S	F	N	D	T	F												40S ribosomal prot. S14
H	W	V	E	F	Q	N	K	F												V-type proton ATPase 116 kDa subunit a isof. 2
Y	Y	T	R	L	G	N	D	F												40S ribosomal prot. S17-like
D	Y	S	S	I	L	Q	K	F												Formin-bind. prot. 1
V	Y	V	V	G	T	A	H	F												TraB domain-containing prot.
R	F	I	G	A	T	A	N	F												Ribonuclease H2 subunit C
E	E	T	I	E	K	M	Q	E												60S ribosomal prot. L26
I	F	T	N	T	V	A	R	F												A-kinase anchor prot. 11
T	Y	G	E	I	F	E	K	F												NADH dehydrogenase [ubiquinone] 1 subunit C2
I	Y	K	Q	D	L	P	S	L												Heat shock prot. 105 kDa
K	F	I	D	T	T	S	K	F												60S ribosomal prot. L3
V	F	P	E	K	G	Y	S	F												Nucleolysin TIAR
P	F	V	D	H	V	F	T	F												Ribosome biogenesis prot. BRX1 homolog
D	Y	A	E	T	V	H	Q	L												Spectrin β chain, brain 1

V Y G P L P Q S F	Cytochrome c oxidase subunit 4 isof. 1, mitochondrial
R Y P T S I A S L	Mitotic checkpoint prot. BUB3
I F T D L S S R F	X-ray repair cross-complementing prot. 5
I Y K A P S E N W	DNA (cytosine-5)-methyltransferase 1
T F A G I E N K F	Calcium-bind. mitochondrial carrier prot. Aralar2
D Y G I V A D L F	Electron transfer flavoprotein subunit α, mitochondrial
R W A P N E N K F	Actin-related prot. 2/3 complex subunit 1B
E E L I E K M Q E	60S ribosomal prot. L26-like 1
I Y Q K P F Q T L	Atlastin-2
A Y V H M V T H F	Bax inhibitor 1
K Y P A S T V Q I	Nucl. prot. 56
I Y G Y V A E Q F	Signal peptidase complex subunit 1
T Y P D V N N S I	Vacuolar prot. sorting-associated prot. 26[a]
V F P A I A Q E I	Cell division control prot. 6 homolog
V F P D K G Y S F	Nucleolysin TIA-1 isof. p40
S Q M K S Y Q S F	General transcription factor 3C polypeptide 1
P Y N A P T V K F	Ubiquitin-conjugating enzyme E2 C
K Y P D I I S R I	DNA ligase 1
I L P K E L Q T W	Zinc finger prot. 24
I F S K I V S L F	Putative eukar. transl. initiation factor 2 subunit 3
V Y I K H P V S L	26S proteasome non-ATPase regulatory subunit 8
E F P S I K T E F	Centromere-associated prot. E
Y Y N K V S T V F	Eukaryotic translation initiation factor 3 subunit A
I D A G Y R H I D	Aldo-keto reductase family 1 member
I Y A P K L Q E F	G1/S-specific cyclin-E2
N Y Y E V H K E L F	Prot. regulator of cytokinesis 1
I Y P E L Q I E R F	Amyloid β A4 precursor prot.-bind. family B member 1
A Y V S R L P A A F	Acyl carrier prot., mitochondrial
H Y P Q F S G Q S F	Arylsulfatase A
T Q P G T G W V Q F	Adaptin ear-bind. coat-associated prot. 2
D Y A A I R D N Y F	Ras-related prot. Ral-A
R F P E E L T Q T F	Elongation factor 1-γ
I Y S T V D D D H F	Replication prot. A 32 kDa subunit
T Y F N G D L K N F	MAP7 domain-containing prot. 3
V L K P G M V V T F	Elongation factor 1-α 1
Y L P N Q L F R T F	ATP-dependent RNA helicase DDX1
P Y A K P I P A Q F	pre-mRNA 3' end processing prot. WDR33
I Y K D L P F E T L	39S ribosomal prot. L39, mitochondrial
A F I R V V G S E F	26S protease regulatory subunit 6B
Y Y P A Q G V Q Q F	Eukaryotic translation initiation factor 4 γ 1
V W A A V P G K T F	Profilin-1

S	F	A	K	I	Y	A	D	T	F		116 kDa U5 small nuclear ribonucleoprot. component
I	Y	P	V	D	L	G	D	K	F		DNA-dir. RNA polymerases I, II, and III subunit RPABC3
L	Y	A	D	V	G	G	K	Q	F		Translocon-associated prot. subunit δ
E	Y	P	D	R	I	M	N	T	F		Tubulin β-2A chain
I	W	I	L	I	D	K	T	S	F		Leukocyte antigen CD37
P	Y	T	D	V	N	I	V	T	I		Isocitrate dehydrogenase [NAD] subunit α, mitochondrial
Y	Y	F	E	G	I	K	Q	T	F		Activator of 90 kDa heat shock prot. ATPase homolog 1
M	Y	K	T	T	P	D	V	I	F		40S ribosomal prot. S24
T	Y	T	D	V	T	P	R	Q	F		StAR-related lipid transfer prot. 7, mitochondrial
H	F	F	Q	P	T	N	Q	Q	F		THO complex subunit 1
V	Y	K	E	N	L	V	D	G	F		Afadin
I	F	T	D	I	G	K	V	D	F		Nucl. pore complex prot. Nup160
R	Y	Y	K	N	I	G	L	G	F		40S ribosomal prot. S11
T	L	P	T	F	Q	S	P	E	F		Sorting nexin-5
V	L	P	E	I	D	P	V	L	F		N-acetyl-D-glucosamine kinase
R	F	P	Q	L	D	S	T	S	F		Fatty acid synthase
S	Y	V	K	L	P	P	E	K	F		Vacuolar prot. sorting-associated prot. 33A
V	F	V	G	N	I	D	D	H	F		Digestive organ expansion factor homolog
N	Y	G	P	M	K	G	G	S	F		Heterogeneous nuclear ribonucleoprot. A3
I	Y	F	P	K	K	E	A	V	T	F	Phospholipase A-2-activating prot.
S	L	P	P	G	L	A	V	K	E	L	60S ribosomal prot. L38
S	F	I	P	S	S	V	P	A	T	F	AP-2 complex subunit β
N	F	F	P	Y	G	D	A	S	K	F	Neuron-specific calcium-bind. prot. hippocalcin
S	K	P	V	F	S	E	S	L	S	D	Mitoch. import inner membr. translocase subunit Tim8 A
T	F	P	N	I	A	S	A	T	K	F	Heme oxygenase 1
A	Y	A	L	G	K	D	F	V	T	L	Sorting nexin-20
L	Y	P	N	I	D	K	D	H	A	F	Signal transducer and activator of transcription 1-α/β
V	W	V	E	E	S	D	K	R	S	F	ATP-dependent RNA helicase DDX3X
A	L	P	A	N	A	I	L	N	Q	F	Polyadenylate-binding prot. 4
I	Y	S	P	D	H	T	N	N	S	F	Transcription factor 4
V	L	P	G	V	D	A	L	S	N	I	Phosphoglycerate kinase 1
T	Y	A	A	L	N	S	K	A	T	F	Ras GTPase-activating-like prot. IQGAP1
E	I	T	A	L	A	P	S	T	M	K	Actin, cytoplasmic 1
V	L	M	T	Q	Q	P	R	P	V	L	H/ACA ribonucleoprot. complex subunit 3
R	Y	I	S	P	D	Q	L	A	D	L	Alpha-enolase
T	Y	V	P	V	T	T	F	K	N	L	60S ribosomal prot. L31
V	Y	V	D	L	G	G	S	H	V	F	Ribosome biogenesis prot. BMS1 homolog
D	Y	V	E	I	N	I	D	H	K	F	Vigilin
S	Y	P	M	V	L	G	A	Q	H	F	E3 ubiquitin-prot. ligase TRIM21
V	F	V	P	R	D	V	P	E	S	F	WD repeat-containing prot. 75
R	F	P	P	Y	H	V	G	Q	T	F	DNA-binding prot. A

V Y S Q I P A A V K L	E3 ubiquitin-prot. ligase UBR5
W Y V H P Q V L Q S F	Chromatin assembly factor 1 subunit A
F Y V P A E P K L A F	60S ribosomal prot. L7
V F I N K G K G F G F	Splicing factor, pro- and gln-rich
R N P P G F A F V E F	Ser/arg-rich splicing factor 3
E P I Y P E V V H M F	Ser/thr-prot. phosph. 2A 56 kDa reg. subunit γ isof.
I Y V N P A N T H Q F	DDB1- and CUL4-associated factor 8
V W I K P G A E Q S F	60S ribosome subunit biogenesis prot. NIP7 homolog
D Y F S E R I K N Q F	Prot. FAM111B
Y W V G E D S T Y K F	60S ribosomal prot. L15
V F I H K D K G F G F	Non-POU domain-containing octamer-bind. prot.
V F I E G A D A E T F	Bifunctional glutamate/proline-tRNA ligase
K Y P E A P P F V R F	Ubiquitin-conjugating enzyme E2 variant 1
A A V V V S K S G S L K	Nucl. and coiled-body phosphoprot. 1
R L P D G T S L T Q T F	UBX domain-containing prot. 1
I Y I D S N N N P E R F	Clathrin hc 1
V F I T K P P D G S A F	Solute carrier family 15 member 4
I L E L A G N A A R D N K	Histone H2A type 2-A
F A D K V P K T A E N F R	Peptidyl-prolyl cis-trans isomer. A
V Y F K P S L T P S G E F	Nucl. pore complex prot. Nup153
Q G S V Q K V Y N G L Q G Y	DnaJ homolog subfamily B member 11
S A D T L W G I Q K E L Q F	L-lactate dehydrogenase A chain
Q A Q N T V T L A T S I K E K	Citrate lyase subunit β-like prot., mitochondrial
H A V S E G T K A V T K Y T S S	Histone H2B type 1-A
S A S S G A E G D V S S E R E P	D-tyrosyl-tRNA(Tyr) deacylase 1
A S N E D V T K A V D I T T P K	PC4 and SFRS1-interacting prot.
H A V S E G T K A V T K Y T S A K	Histone H2B type 1-K
D G R G A L Q N I I P A S T G A A K	Glyceraldehyde-3-phosphate dehydrogenase
T Y F P H F D L S H G S A Q V K G H G	Hemoglobin subunit α
S A A Q A A A Q T N S N A A G K Q L R	Plasminogen activator inhibitor 1 RNA-bind. prot.
S L V S K G T L V Q T K G T G A S G S F	Histone H1.2

Supplementary Table 3:

HLA-B*24:13 derived self peptides

origin: LCL 721.221 cells / Low binding peptides (before TFA)

Peptide position

1	2	3	4	5	6	7	8	9	10	11	12	13	14	15	16	
**Ligands**																**Source**
V	T	E	S	P	A	K	F									Ras association domain-containing prot. 3
P	L	L	L	S	L	L	I									Protocadherin α-9
V	N	S	A	A	H	L	F									Acyl-CoA desaturase
V	F	H	P	S	Q	D	L									Pre-mRNA-processing factor 19
P	Y	V	N	N	V	P	H	L								Methionine--tRNA ligase, cytoplasmic
S	F	S	T	V	H	E	K	F								WD repeat-containing prot. 36
F	Y	P	P	K	V	E	L	F								Multifunctional prot. ADE2
I	Y	A	P	P	L	P	S	L								A-kinase anchor prot. 1, mitochondrial
L	Y	V	D	F	P	Q	H	L								Signal transducer and activator of transcription 6
Q	F	I	G	Y	P	I	T	L								Heat shock prot. HSP 90-α
K	Y	V	K	G	L	I	S	I								WD repeat-containing prot. 35
T	Y	Q	E	V	A	Q	K	F								Adenosine deaminase CECR1
K	F	I	D	T	T	S	K	F								60S ribosomal prot. L3-like
P	Y	N	H	Q	H	E	Y	F								Fatty acid desaturase 2
A	Y	V	H	M	V	T	H	F								Bax inhibitor 1
P	F	L	D	I	Q	K	R	F								NADH dehydrogenase [ubiquinone] iron-sulfur prot. 5
N	Y	V	D	V	D	P	T	F								Pecanex-like prot. 1
K	Y	I	N	T	D	A	K	F								Short transient receptor potential channel 4-associated prot.
D	Y	V	E	G	L	R	V	F								Myosin light polypeptide 6
K	Y	I	S	K	P	E	N	L								Calcium-binding prot. 39
E	Y	L	T	K	V	D	K	L								Clathrin hc 1
V	Y	V	Q	H	P	I	T	F								Lipopolysaccharide-induced tumor necrosis α factor
V	Y	V	E	R	A	E	V	L								BRCA1-associated prot.
N	Y	I	G	L	I	N	R	I								Interferon-induced, double-stranded RNA-activated prot. kinase
P	F	V	D	H	V	F	T	F								Ribosome biogenesis prot. BRX1 homolog
V	Y	E	N	V	S	H	F	L								Mitochondrial folate transporter/carrier
P	Y	L	F	H	V	V	T	F								Neurofibromin
L	Y	P	L	T	N	Y	T	F								Cleavage and polyadenylation specificity factor subunit 5
S	Y	Q	K	V	I	E	L	F								Lymphokine-activated killer T-cell-originated prot. kinase
K	Y	I	A	T	D	F	T	L								Nardilysin
V	F	I	D	K	Q	T	N	L								CUGBP Elav-like family member 2

T	F	S	D	V	E	A	H	F	Zinc finger prot. 280C
I	F	I	D	E	I	D	S	F	ATPase family AAA domain-containing prot. 1
Y	Y	E	E	Q	H	P	E	L	Interleukin-32
N	Y	L	S	H	H	L	T	I	Polyadenylate-bind. prot.-interacting prot. 1
T	Y	G	E	I	F	E	K	F	NADH dehydrogenase [ubiquinone] 1 subunit C2
I	F	S	N	V	R	I	N	L	Caveolin-1
K	Y	F	A	Q	A	L	K	L	Tetratricopeptide repeat prot. 35
I	F	H	E	V	P	L	K	F	NMDA receptor-regulated prot. 2
V	Y	L	D	K	F	I	R	L	Guanine nucleotide-bind. prot.-like 3-like prot.
I	Y	V	H	D	L	L	T	F	Vam6/Vps39-like prot.
K	Y	I	D	Q	K	F	V	L	Ser/thr-prot. phosphatase 2A 56 kDa regulatory subunit δ isof.
A	Y	I	T	Q	K	V	E	F	DEP domain-containing prot. 5
E	Q	I	K	E	Y	H	H	L	E3 ubiquitin-prot. ligase RNF213
N	F	T	N	V	A	A	T	F	ATP-citrate synthase
V	Y	V	V	G	T	A	H	F	TraB domain-containing prot.
T	Y	L	K	A	V	K	L	F	Iron-responsive element-bind. prot. 2
R	Y	V	D	K	V	S	N	L	Probable α-ketoglutarate-dependent dioxygenase
V	V	L	D	D	K	D	Y	F	10 kDa heat shock prot., mitochondrial
S	Y	I	A	L	P	L	T	L	Nodal modulator 1
N	Y	A	R	G	H	Y	T	I	Tubulin α-1A chain
K	Y	I	D	K	T	I	R	V	U6 snRNA-associated Sm-like prot. LSm7
V	Y	E	R	E	L	Q	T	F	Methyltransferase-like prot. 10
V	F	P	E	K	G	Y	S	F	Nucleolysin TIAR
V	Y	I	H	H	F	D	R	I	MOB kinase activator 3a
I	Y	T	S	S	V	N	R	L	Coatomer subunit β
V	Y	L	P	N	I	N	K	I	Rho-related BTB domain-containing prot. 1
K	L	P	S	N	L	P	Q	L	Prot. SDA1 homolog
I	Y	I	D	R	G	V	V	F	Tuftelin-interacting prot. 11
E	Y	I	A	V	V	K	K	L	Acyl-CoA-binding domain-containing prot. 6
N	Y	G	V	L	H	V	T	F	NACHT, LRR and PYD domains-containing prot. 11
Y	Y	I	F	I	P	S	K	F	Probable ATP-dependent RNA helicase DDX47
N	Y	I	H	V	G	A	Q	L	Unconventional myosin-Id
T	Y	L	E	K	A	I	K	I	Ubiquitin carboxyl-terminal hydrolase 7
V	Y	G	P	L	P	Q	S	F	Cytochrome c oxidase subunit 4 isof. 1, mitochondrial
A	Y	L	E	A	H	E	T	F	Nuclear pore complex prot. Nup107
K	Y	L	S	G	I	A	H	F	Mitochondrial-processing peptidase subunit α
V	Y	A	Q	V	A	R	L	F	Paired amphipathic helix prot. Sin3a
D	Y	H	T	K	L	F	L	I	Mediator of RNA polymer. II transcription subunit 16
V	Y	V	K	H	S	I	S	F	AP-3 complex subunit mu-1
K	Y	I	H	S	A	N	V	L	Mitogen-activated prot. kinase 1
K	Y	I	G	E	N	L	Q	L	60S ribosome subunit biogenesis prot. NIP7 homolog

K	Y	P	D	R	V	P	V	I		Gamma-aminobutyric acid receptor-associated prot.
D	V	A	N	K	I	G	I	I		60S ribosomal prot. L23a
S	Y	I	A	H	L	R	Q	L		Musculin
S	Y	I	S	R	T	N	Q	L		Prot. ELYS
N	Y	I	K	D	L	N	I	L		CTD small phosphatase-like prot. 2
G	Y	I	E	R	P	Q	L	I		Poly(A) RNA polymer. GLD2
V	F	V	G	K	G	I	T	F		Cytosol aminopeptidase
D	I	D	K	G	H	V	K	F		ATP-dependent RNA helicase DDX1
R	Y	I	D	R	I	H	I	F		Pre-mRNA-processing-splicing factor 8
E	Y	V	E	K	F	Y	R	I		DNA repair prot. RAD50
I	Y	N	Y	P	E	Q	L	F		T-complex prot. 1 subunit β
K	F	I	D	K	Q	L	E	L		Mitofusin-2
L	Y	T	E	K	F	E	E	F		Alpha-taxilin
K	F	I	S	P	F	T	Q	F		Heparan-sulfate 6-O-sulfotransferase 3
N	Y	K	S	H	H	L	Q	L		CWF19-like prot. 1
S	Y	L	D	V	K	Q	R	L		ATP-bind. cassette sub-family E member 1
H	Y	V	E	K	P	S	T	F		Mannose-1-phosphate guanyltransferase α
T	Y	Q	D	I	Q	N	T	I		DNA-directed RNA polymer. II subunit
G	V	A	V	D	L	I	L	L		Ataxin-10
K	Y	A	R	V	V	Q	K	L		TATA-box-binding prot.
K	Y	I	Q	R	Q	Q	T	I		Interferon-induced GTP-bind. prot. Mx2
V	Y	S	P	H	V	L	N	L		Dynamin-2
L	V	L	D	N	A	F	Y	K		ADAM DEC1
S	Y	M	G	H	F	D	L	L		Splicing factor 3B subunit 5
K	L	K	E	K	T	Y	S	L		Myotubularin-related prot. 6
S	Y	L	T	V	H	K	R	I		Zinc finger prot. 557
L	Y	S	T	I	R	P	Y	L		RRP12-like prot.
L	Y	S	S	K	L	Y	R	F		BTB/POZ domain-containing prot. KCTD20
Q	Y	P	F	H	V	P	L	L		Lymphotoxin-α
V	Y	I	K	H	P	V	S	L		26S proteasome non-ATPase regulatory subunit 8
I	Y	V	I	P	Q	P	H	F		Prot. CASC5
K	Y	V	K	I	F	D	N	F		Zinc finger prot. 195
I	L	K	K	K	S	F	T	F		Pyridine nucleotide-disulfide oxidoreductase domain
Q	Y	L	P	H	V	A	R	L		Surfeit locus prot. 4
H	F	L	G	N	F	K	T	K	F	Phosphatidylinositol 4,5-bisphosphate 3-kinase catal., δ isof.
T	F	V	D	N	I	Q	T	A	F	Integrator complex subunit 1
K	Y	I	F	K	E	R	E	S	F	Activating signal cointegrator 1 complex subunit 1
V	Y	K	D	H	Q	T	I	T	I	DNA replication licensing factor MCM3
K	Y	I	D	Q	G	I	A	E	L	RuvB-like 1
T	Y	S	P	A	L	N	K	M	F	Cellular tumor antigen p53
K	Y	I	T	K	S	F	N	F	Y	Poly(A)-specific ribonuclease PARN

K	Y	L	N	Q	T	S	R	S	F		Squalene synthase
V	F	E	D	P	V	I	S	K	F		28S ribosomal prot. S7, mitochondrial
L	Y	A	D	V	G	G	K	Q	F		Translocon-associated prot. subunit δ
V	Y	A	H	I	Y	H	Q	H	F		MOB kinase activator 1a
A	F	I	R	V	V	G	S	E	F		26S protease regulatory subunit 6B
Y	F	I	T	V	V	P	T	K	L		Endoplasmic reticulum-Golgi intermediate compartment prot. 2
L	Y	L	K	V	K	G	N	V	F		60S ribosomal prot. L19
R	Y	F	D	P	A	N	G	K	F		Elongation factor 2
I	Y	I	S	T	L	K	T	E	F		Activator of 90 kDa heat shock prot. ATPase homolog 1
D	Y	V	P	P	E	L	I	T	L		Translation initiation factor eIF-2B subunit β
I	Y	G	E	K	F	E	D	E	N		Peptidyl-prolyl cis-trans isomer. A
V	Y	N	E	N	L	V	H	M	I		Pre-mRNA-splicing factor SPF27
R	F	L	D	D	L	G	L	K	F		Desmoglein-2
K	Y	I	E	T	T	P	L	T	I		Zinc finger prot. 638
D	Y	L	D	G	V	H	T	V	F		Peptidyl-prolyl cis-trans isomer.-like 4
I	Y	P	V	D	L	G	D	K	F		DNA-directed RNA polymer. I, II, and III subunit RPABC3
A	Y	I	Q	G	L	K	E	K	F		All-trans-retinol 13,14-reductase
T	Y	P	E	G	L	E	V	L	H	F	Centromere prot. J
S	Y	I	R	P	E	D	I	V	N	F	Transmembrane prot. 183A
R	Y	L	P	T	E	Q	E	V	R	F	FAD-dependent oxidoreductase domain-containing prot. 2
K	Y	M	P	N	V	K	V	A	V	F	Spliceosome RNA helicase DDX39B
K	Y	K	N	S	E	I	N	F	S	F	Prot.n phosphatase 1K, mitochondrial
K	Y	L	P	E	N	D	L	K	R	L	Ser/thr-prot. phosphatase 6 catalytic subunit
V	L	P	G	V	D	A	L	S	N	I	Phosphoglycerate kinase 1
I	Y	L	N	H	I	E	P	L	K	I	Thymidylate synthase
V	Y	V	D	L	G	G	S	H	V	F	Ribosome biogenesis prot. BMS1 homolog
I	F	Y	A	G	T	G	N	L	L	L	Coatomer subunit α
T	Y	L	P	A	G	Q	S	V	L	L	Prohibitin
K	Y	L	D	E	D	T	I	Y	H	L	S-adenosylmethionine synthase isof. type-2
R	Y	P	T	S	I	A	S	L	A	F	Mitotic checkpoint prot. BUB3
R	Y	I	S	P	D	Q	L	A	D	L	Alpha-enolase
K	F	I	D	P	K	K	I	K	V	F	ATP-dependent RNA helicase DDX19A
V	F	I	H	K	D	K	G	F	G	F	Non-POU domain-containing octamer-bind. prot.
T	F	P	N	I	A	S	A	T	K	F	Heme oxygenase 1
L	Y	P	N	I	D	K	D	H	A	F	Signal transducer and activator of transcription 1-α/β
V	Y	S	Q	I	P	A	A	V	K	L	E3 ubiquitin-prot. ligase UBR5
R	F	A	H	G	T	A	G	L	V	F	Cytochrome c oxidase assembly prot. COX15 homolog
L	Y	E	E	G	S	N	K	R	L	F	Proteasome subunit α type-3
K	F	F	G	K	E	L	S	T	T	L	Heat shock 70 kDa prot. 4
R	F	V	N	V	V	P	T	F	G	K	40S ribosomal prot. S30
K	Y	I	T	Q	G	Q	L	L	Q	F	Elongation of very long chain fatty acids prot. 5

S	Y	L	E	R	H	G	L	I	N	F						Lys-specific histone demethylase 1a
I	Y	F	P	K	K	E	A	V	T	F						Phospholipase A-2-activating prot.
G	F	G	D	S	R	G	G	G	G	N	F					Heterogeneous nucl. ribonucleoprot. A2/B1
V	S	E	K	G	T	V	Q	Q	A	D	E					60S ribosomal prot. L9
Y	F	I	D	S	T	N	L	K	T	H	F					Zinc finger prot. 593
V	Q	T	K	G	T	G	A	S	G	S	F					Histone H1.1
I	Y	E	E	T	R	G	V	L	K	V	F					Histone H4
V	Y	I	E	S	R	I	G	T	S	T	S	F				Kinesin-like prot. KIF20A
T	F	M	A	A	G	S	S	A	P	E	L	V	T			Sodium/potassium/calcium exchanger 5
A	K	T	D	Q	A	Q	K	A	E	G	A	G	D	A		Non-histone chromosomal prot. HMG-17
I	K	D	D	E	K	E	A	E	E	G	E	D	D	R	D	Heterogeneous nucl. ribonucleoprot. C-like 1

origin: LCL 721.221 cells / High binding peptides (after TFA)

**1**	**2**	**3**	**4**	**5**	**6**	**7**	**8**	**9**	**10**	**11**	**12**	**13**	**14**	**15**	**16**	**17**	**18**	
Y	E	V	S	Q	L	K	D											Cytosolic non-specific dipeptidase
D	E	D	T	I	Y	H	L											S-adenosylmethionine synthase isof. type-2
V	Y	N	E	N	L	V	H											Pre-mRNA-splicing factor SPF27
S	F	S	T	V	H	E	K	F										WD repeat-containing prot. 36
V	F	N	D	V	R	L	L	L										F-actin-capping prot. subunit α-1
K	F	I	D	T	T	S	K	F										60S ribosomal prot. L3-like
L	T	E	A	P	L	N	P	K										Actin, cytoplasmic 1
G	Y	S	N	R	V	V	D	L										Glyceraldehyde-3-phosphate dehydrogenase
A	Y	L	P	V	N	E	S	F										Elongation factor 2
V	F	S	T	V	V	I	H	F										3-hydroxy-3-methylglutaryl-coenzyme A reductase
V	Y	V	Q	H	P	I	T	F										Lipopolysaccharide-induced tumor necrosis α factor
V	Y	V	V	G	T	A	H	F										TraB domain-containing prot.
K	Y	I	T	D	V	V	K	L										Small subunit processome component 20 homolog
V	Y	S	P	H	V	L	N	L										Dynamin-1
Y	Y	E	E	Q	H	P	E	L										Interleukin-32
P	F	V	D	H	V	F	T	F										Ribosome biogenesis prot. BRX1 homolog
L	Y	T	E	K	F	E	E	F										α-taxilin
T	F	S	D	V	E	A	H	F										Zinc finger prot. 280C
N	Y	G	V	L	H	V	T	F										NACHT, LRR and PYD domains-containing prot. 11
A	Y	V	H	M	V	T	H	F										Bax inhibitor 1
V	F	I	D	K	Q	T	N	L										CUGBP Elav-like family member 2
T	Y	I	T	S	V	S	R	L										Oxidative stress-induced growth inhibitor 2
V	Y	V	Q	N	V	V	K	L										AP-3 complex subunit δ-1
K	Y	I	D	K	T	I	R	V										U6 snRNA-associated Sm-like prot. LSm7
T	Y	G	E	I	F	E	K	F										NADH dehydrogenase [ubiquinone] 1 subunit C2

S Y S D P P L K F	Heterogeneous nucl. ribonucleoprot. F
V F T G V V T K L	Cell division cycle and apoptosis regulator prot. 1
P Y V N N V P H L	Methionine--tRNA ligase, cytoplasmic
N Y I H V G A Q L	Unconventional myosin-Id
L V N S A A H L F	Acyl-CoA desaturase
E Y L T K V D K L	Clathrin hc 1
K Y I G E N L Q L	60S ribosome subunit biogenesis prot. NIP7 homolog
G Y I H V T Q T F	Prot. NKG7
K Y I D Q K F V L	Ser/thr-prot. phosphatase 2A 56 kDa regul. subunit δ isof.
A Y L E A H E T F	Nucl. pore complex prot. Nup107
Q Y V D F H N Q L	DNA repair prot. complementing XP-G cells
S Y L D R T E Q L	WD repeat-containing prot. 18
K Y I Q R Q E T I	Interferon-induced GTP-binding prot. Mx1
I Y G E K T Y A F	Serpin B10
K Y L S G I A H F	Mitochondrial-processing peptidase subunit α
K Y Q E V T N N L	Caprin-1
T Y L E K A I K I	Ubiquitin carboxyl-terminal hydrolase 7
I Y I K Q I K T F	Retinoblastoma-like prot. 2
K Y T P P P H H I	NADH dehydrogenase [ubiquinone] iron-sulfur prot. 5
R Y G L V T N E I	Interferon-induced helicase C domain-containing prot. 1
K Y L N Q T S R S F	Squalene synthase
A Y V K G G L S T F	Exocyst complex component 2
Y Y F E G I K Q T F	Activator of 90 kDa heat shock prot. ATPase homolog 1
I F L P I L R T G F	DNA replication licensing factor MCM7
V L L A P G F Q I P P	Dynein heavy chain 17, axonemal
I L E L A G N A A R D	Histone H2A type 2-A
I Y E D P Q H H P L L	DDB1- and CUL4-associated factor 7
T F P N I A S A T K F	Heme oxygenase 1
R Y I S P D Q L A D L	α-enolase
I Y V N P A N T H Q F	DDB1- and CUL4-associated factor 8
T Y L P A G Q S V L L	Prohibitin
K F F G K D I S T T L	Heat shock prot. 105 kDa
R Y P T S I A S L A F	Mitotic checkpoint prot. BUB3
R Y L P P A T Q V V L	Eukaryotic initiation factor 4A-III
I K P S V E P S A G H D E L	Stromal cell-derived factor 2-like prot. 1
Q V T Q P T V G M N F K T P R G P V	40S ribosomal prot. S17-like

origin: LCL 721.220 cells / Low binding peptides (before TFA)

1	2	3	4	5	6	7	8	9	10	11	12	13	14	
I	Y	D	P	N	L	A	F							1-phosphatidylinositol 4,5-bisphosphate phosphodiesterase γ-2
S	A	R	I	Q	D	A	L							Eukaryotic translation initiation factor 3 subunit F
I	S	P	H	G	N	A	L							ATP-depend. Clp protease ATP-bind. subunit clpX-like
F	E	G	P	P	F	K	F							WD repeat-containing protein 1
I	Y	N	E	A	L	K	G							Prot. S100-A6
I	Y	A	P	P	L	P	S	L						A-kinase anchor prot. 1, mitochondrial
I	F	K	D	K	P	I	K	L						SH3 domain-containing kinase-bind. prot. 1
V	F	L	R	A	I	N	K	F						Dedicator of cytokinesis prot. 2
T	Y	L	E	K	A	I	K	I						Ubiquitin carboxyl-terminal hydrolase 7
I	Y	I	K	H	P	H	L	F						Deoxynucleotidyltransferase terminal-interacting prot. 1
V	Y	P	D	G	I	R	H	I						Splicing factor 3B subunit 3
V	Y	V	K	H	S	I	S	F						AP-3 complex subunit mu-1
L	I	D	R	P	A	P	H	F						Band 4.1-like prot. 2
V	G	P	P	G	R	S	S	L						Nuclear pore complex prot. Nup93
N	Y	I	D	K	V	R	F	L						Vimentin
Y	Y	I	F	I	P	S	K	F						Probable ATP-dependent RNA helicase DDX47
T	Y	H	P	G	V	P	V	F						Cys and his-rich domain-containing prot. 1
K	Y	I	S	K	P	E	N	L						Calcium-bind. prot. 39
F	Y	I	S	P	V	N	K	L						Late secretory pathway prot. AVL9 homolog
L	Y	T	E	K	F	E	E	F						α-taxilin
L	V	N	S	A	A	H	L	F						Acyl-CoA desaturase
S	F	S	T	V	H	E	K	F						WD repeat-containing prot. 36
D	Y	V	E	G	L	R	V	F						Myosin light polypeptide 6
I	Y	G	E	K	T	Y	A	F						Serpin B10
T	F	S	D	V	E	A	H	F						Zinc finger prot. 280C
V	Y	Q	H	L	F	T	R	I						AP-5 complex subunit ζ-1
K	F	I	D	T	T	S	K	F						60S ribosomal prot. L3-like
I	L	T	E	R	G	Y	S	F						Actin, cytoplasmic 1
I	Y	I	A	G	H	P	A	F						Heterogeneous nucl. ribonucleoprot. L
V	F	I	D	K	Q	T	N	L						CUGBP Elav-like family member 2
A	Y	V	H	M	V	T	H	F						Bax inhibitor 1
V	Y	V	V	G	T	A	H	F						TraB domain-containing prot.
T	Y	G	E	I	F	E	K	F						NADH dehydrogenase [ubiquinone] 1 subunit C2
V	Y	S	P	H	V	L	N	L						Dynamin-1
V	F	T	G	V	V	T	K	L						Cell division cycle and apoptosis regulator prot. 1
D	Y	G	I	V	A	D	L	F						Electron transfer flavoprotein subunit α, mitochondrial
K	Y	I	N	T	D	A	K	F						Short transient receptor potential channel 4-associated prot.
V	Y	G	P	L	P	Q	S	F						Cytochrome c oxidase subunit 4 isof. 1, mitochondrial

H Y V P A T K V F	L-amino-acid oxidase
P F L D I Q K R F	NADH dehydrogenase [ubiquinone] iron-sulfur prot. 5
P F V D H V F T F	Ribosome biogenesis protein BRX1 homolog
V Y I K H P V S L	26S proteasome non-ATPase regulatory subunit 8
P F L P K P L F F	Ribosomal RNA processing prot. 1 homolog B
E Y I A V V K K L	Acyl-CoA-binding domain-containing prot. 6
I Y I K Q I K T F	Retinoblastoma-like prot. 2
F Y P P K V E L F	Multifunctional prot. ADE2
N Y G V L H V T F	NACHT, LRR and PYD domains-containing prot. 11
I Y V H D L L T F	Vam6/Vps39-like prot.
Y Y E E Q H P E L	Interleukin-32
E F P S I K T E F	Centromere-associated prot. E
K Y I D Q K F V L	Ser/thr-prot. phosphatase 2A 56 kDa regul.subunit δ isof.
Y Y K N I G L G F	40S ribosomal prot. S11
S Y I S R T N Q L	Prot. ELYS
V Y E R E L Q T F	Methyltransferase-like prot. 10
E Y L K I K Q S F	E3 ubiquitin-prot. ligase RNF180
P F I D S Q H V I	Dolichol phosphate-mannose biosynthesis regulatory prot.
I Y Q R H V Y N L	Transmembrane prot. 209
P Y V N N V P H L	Methionine-tRNA ligase, cytoplasmic
K Y I D K T I R V	U6 snRNA-associated Sm-like prot. LSm7
E F I N T T A R V	Lactoperoxidase
V A E E H L T V D	ADP-sugar pyrophosphatase
I F H E V P L K F	NMDA receptor-regulated prot. 2
I L K K K S F T F	Pyridine nucleot.-disulf. oxidoreduct. prot. 1
T Y Q D I Q N T I	DNA-directed RNA polymerase II subunit RPB1
K Y I S G P H E L	Centromere prot. F
T Y V S G T L R F	Choline/ethanolaminephosphotransfer. 1
N F T N V A A T F	ATP-citrate synthase
S T L D P K T I S F	Acyl-coenzyme A thioesterase 9, mitochondrial
T I I G E S I S R L	Arginine-tRNA ligase, cytoplasmic
Y Y F E G I K Q T F	Activator of 90 kDa heat shock prot. ATPase homolog 1
L Y A D V G G K Q F	Translocon-associated prot. subunit δ
V Y V Q I H P I T F	Lipopolysaccharide-induced tumor necrosis α factor
R Y F D P A N G K F	Elongation factor 2
V F E D P V I S K F	28S ribosomal prot. S7, mitochondrial
I F L P I L R T G F	DNA replication licensing factor MCM7
L Y L K V K G N V F	60S ribosomal prot. L19
A F I R V V G S E F	26S protease regulatory subunit 6B
V Y V D L G G S H V F	Ribosome biogenesis prot. BMS1 homolog
H Y F Q N T Q G L I F	ADP-ribosylation factor 1
T F P N I A S A T K F	Heme oxygenase 1

T	Y	L	P	A	G	Q	S	V	L	L				Prohibitin
K	F	L	D	A	G	H	K	L	N	F				Prot. disulfide-isomer. A3
V	F	I	H	K	D	K	G	F	G	F				Non-POU domain-containing octamer-bind. prot.
K	F	F	G	K	D	I	S	T	T	L				Heat shock prot. 105 kDa
K	Y	L	D	E	D	T	I	Y	H	L				S-adenosylmethionine synthase isof. type-2
R	Y	P	T	S	I	A	S	L	A	F				Mitotic checkpoint prot. BUB3
R	F	L	N	A	E	N	A	Q	K	F				Ran-specific GTPase-activating prot.
V	L	P	E	G	G	E	T	P	L	F				Gelsolin
G	V	L	K	P	G	M	V	V	T	F				Elongation factor 1-α 1
R	Y	I	S	P	D	Q	L	A	D	L				Alpha-enolase
T	Y	V	P	V	T	T	F	K	N	L				60S ribosomal prot. L31
R	F	V	N	V	V	P	T	F	G	K				40S ribosomal prot. S30
I	Y	E	D	P	Q	H	H	P	L	L				DDB1- and CUL4-associated factor 7
F	Y	V	P	A	E	P	K	L	A	F				60S ribosomal prot. L7
G	Y	Q	R	D	G	Y	Q	Q	N	F				Caprin-1
L	Y	P	N	I	D	K	D	H	A	F				Signal transducer and activator of transcription 1-α/β
Y	F	I	D	S	T	N	L	K	T	H	F			Zinc finger prot. 593
I	Y	I	D	S	N	N	N	P	E	R	F			Clathrin hc 1
N	F	G	F	G	D	S	R	G	G	G	G	N	F	Heterogeneous nucl. ribonucleoprot. A2/B1

origin: LCL 721.220 cells / High binding peptides (after TFA)

**1**	**2**	**3**	**4**	**5**	**6**	**7**	**8**	**9**	**10**	**11**	**12**	**13**	**14**	**15**	
F	V	Q	M	M	T	A	K								Calmodulin
L	V	D	V	E	P	K	V								Small nuclear ribonucleoprot. Sm D1
G	Y	S	N	R	V	V	D	L							Glyceraldehyde-3-phosphate dehydrogenase
K	F	I	D	T	T	S	K	F							60S ribosomal prot. L3-like
Y	Y	E	E	Q	H	P	E	L							Interleukin-32
V	F	I	D	K	Q	T	N	L							CUGBP Elav-like family member 2
L	Y	E	V	S	Q	L	K	D							Cytosolic non-specific dipeptidase
A	Y	V	H	M	V	T	H	F							Bax inhibitor 1
T	Y	G	E	I	F	E	K	F							NADH dehydrogenase [ubiquinone] 1 subunit C2
V	Y	I	K	H	P	V	S	L							26S proteasome non-ATPase regulatory subunit 8
V	F	V	D	R	T	L	D	L							Sec1 family domain-containing prot. 2
N	V	E	W	A	K	P	S	T	N						Eukaryotic translation initiation factor 3 subunit G
I	L	E	L	A	G	N	A	A	R	D					Histone H2A type 2-A
A	A	L	E	K	E	A	Q	T	S	F					ATP-dependent RNA helicase DDX1
R	Y	I	S	P	D	Q	L	A	D	L					Alpha-enolase
T	Y	V	P	V	T	T	F	K	N	L					60S ribosomal prot. L31
F	Q	I	N	Q	D	E	E	E	E	E	D	E	D		60S ribosomal prot. L22
N	F	G	F	G	D	S	R	G	G	G	G	N	F		Heterogeneous nuclear ribonucleoprot. A2/B1

I K P S V E P S A G H D E L	Stromal cell-derived factor 2-like prot. 1
Y A S G R T T G I V M D S G D	Actin, cytoplasmic 1
L V S N L N P E R V T P Q S L	Polypyrimidine tract-bind. prot. 1
